# Safety and Immunogenicity of Radiation-Attenuated PfSPZ Vaccine in Equatoguinean Infants, Children, and Adults

**DOI:** 10.4269/ajtmh.22-0773

**Published:** 2023-05-09

**Authors:** Said A. Jongo, Vicente Urbano Nsue Ndong Nchama, L. W. Preston Church, Ally Olotu, Stephen R. Manock, Tobias Schindler, Ali Mtoro, Natasha KC, Orrin Devinsky, Elcin Zan, Ali Hamad, Elizabeth Nyakarungu, Maxmillian Mpina, Anna Deal, José Raso Bijeri, Martin Eka Ondo Mangue, Beltrán Ekua Ntutumu Pasialo, Genaro Nsue Nguema, Matilde Riloha Rivas, Mwajuma Chemba, Kamaka K. Ramadhani, Eric R. James, Thomas C. Stabler, Yonas Abebe, Pouria Riyahi, Elizabeth S. Saverino, Julian Sax, Salome Hosch, Anneth Tumbo, Linda Gondwe, J. Luis Segura, Carlos Cortes Falla, Wonder Philip Phiri, Dianna E. B. Hergott, Guillermo A. García, Carl Maas, Tooba Murshedkar, Peter F. Billingsley, Marcel Tanner, Mitoha Ondo’o Ayekaba, B. Kim Lee Sim, Claudia Daubenberger, Thomas L. Richie, Salim Abdulla, Stephen L. Hoffman

**Affiliations:** ^1^ Ifakara Health Institute, Bagamoyo Research and Training Centre, Bagamoyo, Tanzania;; ^2^ Ministry of Health and Social Welfare, Government of Equatorial Guinea, Malabo, Equatorial Guinea;; ^3^ Sanaria Inc., Rockville, Maryland;; ^4^ Swiss Tropical and Public Health Institute, Allschwil, Switzerland;; ^5^ University of Basel, Basel, Switzerland;; ^6^ Protein Potential LLC, Rockville, Maryland;; ^7^ New York University Langone Medical Center, New York, New York;; ^8^ MCD Global Health, Silver Spring, Maryland;; ^9^ Marathon EG Production, Ltd., Malabo Dos, Equatorial Guinea

## Abstract

The radiation-attenuated *Plasmodium falciparum* sporozoites (PfSPZ) Vaccine has demonstrated safety and immunogenicity in 5-month-old to 50-year-old Africans in multiple trials. Except for one, each trial has restricted enrollment to either infants and children or adults < 50 years old. This trial was conducted in Equatorial Guinea and assessed the safety, tolerability, and immunogenicity of three direct venous inoculations of 1.8 × 10^6^ or 2.7 × 10^6^ PfSPZ, of PfSPZ Vaccine, or normal saline administered at 8-week intervals in a randomized, double-blind, placebo-controlled trial stratified by age (6–11 months and 1–5, 6–10, 11–17, 18–35, and 36–61 years). All doses were successfully administered. In all, 192/207 injections (93%) in those aged 6–61 years were rated as causing no or mild pain. There were no significant differences in solicited adverse events (AEs) between vaccinees and controls in any age group (*P* ≥ 0.17). There were no significant differences between vaccinees and controls with respect to the rates or severity of unsolicited AEs or laboratory abnormalities. Development of antibodies to *P. falciparum* circumsporozoite protein occurred in 67/69 vaccinees (97%) and 0/15 controls. Median antibody levels were highest in infants and 1–5-year-olds and declined progressively with age. Antibody responses in children were greater than in adults protected against controlled human malaria infection. Robust immunogenicity, combined with a benign AE profile, indicates children are an ideal target for immunization with PfSPZ Vaccine.

## INTRODUCTION

Despite global investment of $2.7–$4.3 billion annually in malaria control, malaria cases and deaths were stable from 2015 to 2019^1^ and increased in 2020.^2^ On Bioko Island, Equatorial Guinea, malaria prevalence has been constant at 10.5–12.7% in 2–14-year-old children since 2012,^3^^,^^4^ despite ongoing deployment of bed nets, indoor residual spraying, and case detection and treatment. The annual investment to maintain this status quo on Bioko Island is nearly $30 U.S. per capita.

A safe and effective malaria vaccine with sustained immunity across all age groups would be the most efficient way to decrease transmission and eliminate malaria from Bioko Island.^4^ PfSPZ Vaccine, which consists of purified, radiation-attenuated *Plasmodium falciparum* sporozoites (PfSPZ), has shown excellent safety in adults without^5^^–^^9^ and with^10^^–^^15^ prior malaria exposure and in infants and children residing in endemic *P. falciparum*–endemic.^13^^,^^16^^,^^17^ It provides up to 100% protection against controlled human malaria infection (CHMI)^5^^,^^14^^,^^18^ and had vaccine efficacies of 42–61% over 6–18 months against *P. falciparum* infection in four African field trials in which clearance of existing parasitemia was performed prior to vaccination.^10^^,^^18^^–^^20^

This study evaluated the safety and immunogenicity of PfSPZ Vaccine in Equatoguinean participants aged 6 months to 61 years. It is the first trial to evaluate the safety of PfSPZ Vaccine in older adults and the second to provide a direct comparison of adverse events (AEs) and immunogenicity across multiple age strata.^13^

## MATERIALS AND METHODS

### Study design and population.

This single-center, double-blind, randomized, placebo-controlled trial of PfSPZ Vaccine was conducted in Baney, Equatorial Guinea, between October 10, 2016 and June 29, 2018 (last study participant visit: January 10, 2018). It had two major components after initial dosing in 18–35-year-old adults: an age de-escalation component to assess safety and immunogenicity in Equatoguinean children and infants and an age escalation component to assess safety and immunogenicity in older adults. In a substudy, a second group of 18–35-year-old adults was immunized with PfSPZ-CVac (non-attenuated PfSPZ attenuated in vivo by coadministration of chloroquine), and the two young adult groups immunized with PfSPZ Vaccine and PfSPZ-CVac underwent CHMI to assess vaccine efficacy (VE).^14^ The safety and immunogenicity of PfSPZ Vaccine in all age groups is described in this report (see Jongo et al.^14^ for data on the safety, immunogenicity, and protective efficacy of PfSPZ-CVac).

Healthy male and female participants aged 6 months to 65 years were recruited from the Baney District and the city of Malabo on Bioko Island. Participants who met the inclusion and exclusion criteria (Supplemental Appendix) were consented and enrolled after they or their parents successfully completed a test of understanding. In addition to consent from the parents, written (for ages 11–17 years) or verbal (ages 6–10 years) assent was obtained from children in these age groups. Eligibility criteria are available at https://clinicaltrials.gov/show/NCT02859350.

### Intervention and randomization.

Participants were allocated to six age groups (Groups 1, 2, 3, 4, 5, and 6b; Supplemental Table 1) and were randomized to receive either three doses of PfSPZ Vaccine (1.8 × 10^6^ PfSPZ for age < 18 years and 2.7 × 10^6^ PfSPZ for age ≥ 18 years) or normal saline (NS) as a placebo by direct venous inoculation (DVI) on days 1, 57, and 113. A pilot group of three participants aged 6–11 months (Group 6a) was immunized with a single dose of 9.0 × 10^5^ PfSPZ of PfSPZ Vaccine to evaluate safety prior to randomization of the remaining infant participants to receive 1.8 × 10^6^ PfSPZ or placebo. The number of participants planned for enrollment for Groups 2, 3, 4, 5, and 6b was 16 each, with 12 receiving PfSPZ Vaccine and 4 receiving NS; in contrast, in Group 1 (adults 18–35 years old), 26 participants were enrolled, with 20 receiving PfSPZ Vaccine and 6 receiving NS.

### Investigational products.

Sanaria^®^ PfSPZ Vaccine is composed of live (metabolically active) radiation-attenuated, aseptic, purified PfSPZ cryopreserved in liquid nitrogen vapor phase at −150 to −196°C.^21^ Preparation of investigational products in 0.5 mL was done under the supervision of the unblinded study pharmacist. PfSPZ Vaccine or NS at 0.5 mL was administered by DVI through a 25-gauge needle by blinded clinical staff.

### Adverse events.

Solicited local AEs were collected for 3 days after each immunization. These were pain, tenderness, pruritus, erythema, swelling, induration, and bruising/extravasated blood (Supplemental Table 2). Solicited systemic and unsolicited AEs were collected for 7 and 28 days, respectively, after each immunization. Solicited systemic AEs were headache, subjective fever, fatigue, malaise, chills, myalgia, arthralgia, objective fever, rash, urticaria, pruritus, and edema for children aged 6 years and older, adolescents, and adults and subjective fever, drowsiness, irritability/fussiness, inability/refusal to eat or drink, objective fever, rash, urticaria, pruritus, and edema for infants and children aged 5 years or younger (Supplemental Table 2). Participants were observed for 2 hours after administration of PfSPZ Vaccine, then followed with daily home or clinic visits. Any participant who reported AEs at home was referred to the clinic for further evaluation. Solicited and unsolicited AEs were recorded and graded by physicians as mild (easily tolerated), moderate (interfering with normal activity), or severe (preventing normal activity). Axillary temperature was categorized as grade 1 (38.0–38.4°C), grade 2 (38.5–38.9°C), or grade 3 (≥ 39.0°C) (Supplemental Table 2). All AEs were additionally assessed as definitely, probably, or possibly related to PfSPZ Vaccine administration (collectively considered “related”) or unlikely or not related (collectively considered “unrelated”).

Hematological and biochemical abnormalities were assessed separately from AEs using standard clinical assays and were graded according to a predetermined severity scale (Supplemental Table 3). The clinical significance of abnormal measurements was assessed in the context of the overall health of the participant, including the timing of the most recent immunization and recent or active AEs.

### Detection of *P. falciparum* parasites and treatment of study participants.

Samples for preparation of thick blood smears (TBS) and analysis by quantitative polymerase chain reaction (qPCR) were obtained within 24 hours before each immunization and at any time a participant was suspected of having symptomatic malaria. Only the TBS was used in real time for diagnosis of malaria (excluding participation in CHMI by adults 18–35 years of age and reported in Jongo et al.^14^). Slide preparation and reading for TBSs were performed as previously described.^22^ In brief, 10 µL of blood collected in ethylenediaminetetraacetic acid (EDTA) was placed on a 10 mm by 20 mm rectangle on a glass slide, dried, and stained with Giemsa stain. The presence of two or more asexual erythrocytic-stage *P. falciparum* parasites in five passes (∼0.5 µL of blood), confirmed by the reading of a second microscopist blinded to the results of the first microscopist, was considered positive. *Plasmodium* sp. infections diagnosed in participants prior to the CHMI (Group 1) or during the course of study participation (Groups 2–6) were treated according to national guidelines with artesunate-amodiaquine or artemether-lumefantrine. Samples obtained for qPCR were analyzed retrospectively using the PlasQ qPCR assay as described.^23^ The lower limit of detection for this qPCR assay was 50 parasites/mL.

### Antibody assays.

Blood for immunogenicity testing was drawn prior to the first immunization and 2 weeks after the final immunization. Serum was separated and frozen at −80°C within 4 hours of collection. Immunoglobulin G antibodies to *P. falciparum* circumsporozoite protein (PfCSP) were assessed by ELISA as described.^13^^,^^24^ The serum dilution at which the optical density (OD) was 1.0 (OD 1.0) was reported, as well as the difference between post-vaccination OD 1.0 and pre-vaccination OD 1.0 (net OD 1.0). The ratio of post–OD 1.0 to pre–OD 1.0 (OD 1.0 ratio) was calculated. An individual was considered to have seroconverted if the net OD 1.0 was ≥ 50 and the OD 1.0 ratio was ≥ 3.0.

### Statistical analysis.

Sample sizes of 12 vaccinees and 4 controls in each age group were selected to be appropriate for the initial assessment of safety, tolerability, and immunogenicity of an investigational vaccine; a larger sample size of 20 vaccinees and 6 NS controls was selected for adults aged 18–35 years on the basis of power calculations for efficacy in a CHMI study.^14^ Categorical variables were summarized using absolute (*N*) and relative (%) frequencies. Continuous variables were summarized using mean and SD, median, and range. Comparisons of categorical variables between groups were analyzed using a two-tailed Fisher’s exact test; for comparisons of continuous variables, the Mann-Whitney two-sided test was used. For PfCSP antibody measurements, we analyzed differences between vaccinees and controls using two-tailed Fisher’s exact test for seroconversion rates, the Mann-Whitney test for net OD 1.0 and OD 1.0 ratios, and nonparametric analysis of variance for comparing net OD and OD ratios between groups. No corrections were made for multiple comparisons because of the early phase nature of this trial. A *P* value < 0.05 was considered significant.

## RESULTS

One hundred and eighty-eight adults, children, and infants were successfully screened, from whom 106 participants were enrolled and received injections. Age groups were enrolled sequentially in the following order (for safety reasons): 18–35 years, 36–61 years, 11–17 years, 6–10 years, 1–5 years, and 6–11 months (Supplemental Table 1). The demographics within each group were similar between vaccine and NS recipients (Supplemental Table 4). Screening and enrollment were straightforward for all groups < 18 years of age, with only 15 screening failures. Thirty-two 18–35-year-olds did not meet the inclusion/exclusion criteria, predominantly for positive serologic testing for HIV, hepatitis B, or hepatitis C (12) or for failing to meet body mass index (BMI) criteria (7); thirty-five 36–61-year-olds did not meet the inclusion/exclusion criteria, predominantly for chronic disease (12) or for failing to meet BMI criteria (8) (Supplemental Table 5).

### Success and tolerability of DVI.

**Figure 1.**
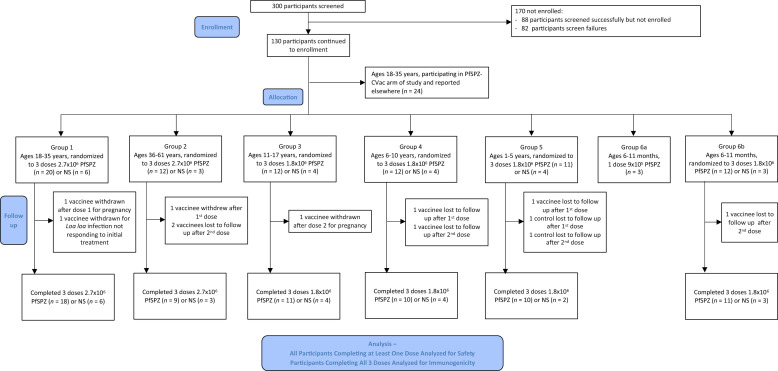
CONSORT diagram—study enrollment, group allocation, and analysis populations. CVac = non-attenuated PfSPZ attenuated in vivo by coadministration of chloroquine; NS = normal saline; PfSPZ = *Plasmodium falciparum* sporozoites.

One hundred four of 106 participants aged 6 months to 61 years were administered study product by DVI: 80 participants received 223 doses of PfSPZ Vaccine by DVI, and 24 participants received 69 doses of NS (Figure 1). Two infants in the pilot arm received a single dose of vaccine through an intravenous cannula, an option made available to the study team when anticipating difficult intravenous access. All participants in the vaccine and NS arms were successfully injected. Direct venous inoculation was successful with the first attempt in 267 of 292 injections (91%) overall, ranging from 95% (196 of 207) in the 6–10, 11–17, 18–35, and 36–61 year age groups to 85% (34 of 40) in the 1–5-year-old group and 80% (36 of 45) in the 6–11-month-old group (*P* = 0.0004 for the comparison of all groups, χ^2^) (Supplemental Table 6). Pain with DVI was rated as mild or none in 145 of 156 PfSPZ Vaccine doses (93%) and 47 of 51 NS doses (92%) in participants aged 6 years and above (Supplemental Table 6).

### Safety.

#### Solicited AEs.

When all age groups were combined, solicited local AEs were observed with 12 of 225 doses of PfSPZ Vaccine (5.3%) compared with 2 of 69 doses of NS (2.9%; *P* = 0.74, Fisher’s exact test, two-tailed) (Table 1), and solicited systemic AEs were observed with 39 of 225 doses of PfSPZ Vaccine (17.3%) compared with 7 of 69 doses of NS (10.1%; *P* = 0.25, Fisher’s exact test, two-tailed) (Table 1). Within each age group, there were no significant differences between vaccinees and controls in the number of doses associated with at least one solicited local or at least one solicited systemic AE (*P* > 0.27 for all comparisons, Fisher’s exact test, two-tailed). Of 13 local solicited AEs (pain, tenderness, or pruritus) recorded in vaccinees, 11/13 were grade 1 or not graded and 2/13 were grade 2. Three local solicited AEs were recorded in the controls, with 3/3 grade 1 or not graded.

**Table 1:** Solicited adverse events per vaccine dose, by group

	Group 1 2.7 × 10^6^ PfSPZ Age 18–35 years		Group 2 2.7 × 10^6^ PfSPZ Age 35–65 years		Group 3 1.8 × 10^6^ PfSPZ Age 11–17 years		Group 4 1.8 × 10^6^ PfSPZ Age 6–10 years		Group 5 1.8 × 10^6^ PfSPZ Age 1–5 years		Groups 6a/6b 9 × 10^5^/1.8 × 10^6^ PfSPZ Age 6–11 months	
Adverse event	Vaccine *N* = 56	NS *N* = 18	Vaccine *N* = 32	NS *N* = 9	Vaccine *N* = 35	NS *N* = 12	Vaccine *N* = 33	NS *N* = 12	Vaccine *N* = 31	NS *N* = 9	Vaccine *N* = 38	NS *N* = 9
Any solicited AE	10 (18%)	1 (5.5%)	8 (25%)	2 (22%)	10 (29%)	4 (33%)	6 (18%)	0	5 (16%)	0	8 (21%)	2 (22%)
Any solicited local AE*	0	0	1 (3.1%)	0	4 (11%)	2 (17%)	1 (3.0%)	0	3 (9.7%)	0	1 (2.6%)	0
Any solicited systemic AE†	10 (18%)	1 (5.5%)	7 (22%)	2 (22%)	6 (17%)	2 (17%)	5 (15%)	0	3 (9.7%)	0	7 (18%)	2 (22%)
Headache	3	0	5	2	6	0	3	0	–	–	–	–
Fatigue	5	0	0	1	0	0	0	0	–	–	–	–
Myalgia	3	0	1	0	0	1	0	0	–	–	–	–
Arthralgia	3	1	1	1	1	0	0	0	–	–	–	–
Allergic reaction†	0	0	0	0	0	0	1	0	1	0	1	0
Subjective fever	3	0	1	1	1	1	2	0	2	0	5	2
T ≥ 38.0°C‡	0	0	0	0	0	0	0	0	1	0	3	0

AE = adverse event; PfSPZ = *Plasmodium falciparum* sporozoites. For each group, *N* = the number of doses administered to the participants in that group; T = temperature (axillary). Data are presented as absolute number of injections with events and as the percentage of all injections. Within each group, there were no significant differences in AEs between the vaccinees and controls for any AE (*P* > 0.27 for all comparisons, Fisher’s exact test, two-tailed).

* Solicited local AEs included pain, tenderness, pruritus, erythema, induration, swelling, and bruising.

† Solicited systemic AEs included fever, subjective fever, and allergic reaction (defined as urticaria or a rash, swelling, or pruritus distant from the site of injection) for all participants. Additional observations in participants aged 6 years and older included headache, myalgias, arthralgias, fatigue, malaise, and chills. In participants < 5 years old, solicited systemic AEs included drowsiness, irritability/fussiness, or inability or refusal to eat or drink.

‡ Maximum observed temperature in any participant was 38.5°C.

The most frequent solicited systemic AE was headache, associated with 17 of 156 PfSPZ Vaccine doses in 15/56 participants aged 6–61 years (26.7%) compared with 2 of 51 NS doses in 1/17 participants (5.9%), a difference that was not significant (*P* = 0.17/0.10 for number of events and number of individuals). For the two participants who experienced headache after more than one dose, the severity with the subsequent dose remained mild for one participant and increased from mild to severe for the second participant (described below). There were no significant differences in rates of fatigue, myalgias, or arthralgias between vaccinees and controls aged 6–61 years (*P* ≥ 0.68) and no significant differences in the rates of subjective fever or elevated temperature (*P* ≥ 0.58) for participants aged 6 months to 61 years. All solicited AEs were considered related to immunization. Of the 52 systemic solicited AEs recorded in vaccinees, 44/52 were grade 1 or not graded, 6/52 were grade 2, and 2/52 were grade 3 (described below). Of the 12 systemic solicited AEs in controls, 11/12 were grade 1 and 1/12 was grade 2 in severity.

One participant, a 7-year-old girl, developed mild (grade 1) generalized pruritus without a visible rash 4 days after her first dose of 1.8 × 10^6^ PfSPZ. This was assessed as a possible allergic reaction, and she was subsequently excluded from further immunizations. No participant reported urticaria or symptoms suggestive of angioedema or anaphylaxis.

Two grade 3 (severe) systemic solicited AEs were reported. A 15-year-old female experienced headache starting 1 day after her second immunization with 1.8 × 10^6^ PfSPZ. She had reported mild headache after her first immunization. This episode was initially moderate but was severe by the third day. She was treated with metamizole (Novalgin^®^) with resolution by the following day. This participant subsequently became pregnant and did not receive a third vaccine dose. A 15-year-old male experienced an episode of grade 3 headache after his third dose of 1.8 × 10^6^ PfSPZ. This participant had a history of recurring headaches for approximately 1 year prior to enrollment into the study, although in this case the headache occurred after the participant experienced a solitary generalized seizure 3.5 hours after immunization (described further under serious adverse events). This headache resolved in the next 48 hours. All other solicited AEs were grade 2 or less.

#### Unsolicited AEs.

During the immunization period, 114 unsolicited AEs were reported across all age groups receiving PfSPZ Vaccine (85 occurrences) or saline control (29 occurrences). Six unsolicited AEs (four grade 1 and two grade 2) in six participants (7.3%) were considered possibly related to the administration of 1.8 × 10^6^ or 2.7 × 10^6^ PfSPZ; two unsolicited AEs (both grade 2) in two participants (8.3%) were considered possibly related to the administration of NS (Supplemental Table 7). Three unsolicited, unrelated AEs were grade 3 in severity, two occurring in vaccinees (toothache, stomatitis) and one in a saline control (malaria).

#### Pregnancy.

Despite vigorous counseling regarding the use of contraception, three participants became pregnant during the study as described below (further details of each pregnancy are included in the Supplemental Appendix). One of these events was classified as a possibly related serious adverse event (SAE; spontaneous abortion) and led to study pause.

1. An 18-year-old woman was hospitalized for hyperemesis gravidarum. Symptom onset was 19 weeks after her last dose of PfSPZ Vaccine. The remainder of her pregnancy was uneventful, and she delivered a healthy girl at 37½ weeks.
2. A previously healthy 15-year-old had a positive pregnancy test 52 days after her second dose of 1.8 × 10^6^ PfSPZ of PfSPZ Vaccine. Last menstrual period and estimated date of conception by ultrasound were 32 and 41 days after immunization, respectively. The pregnancy proceeded normally until week 29, when intrauterine growth restriction was diagnosed by ultrasound. The mother developed hypertension, mild edema, and 2+ proteinuria, leading to delivery of a 1,300-gram infant by cesarean section at 33 weeks. The infant was subsequently found to have a patent ductus arteriosus, moderate tricuspid insufficiency, and a patent foramen ovale, all of which were managed medically. A protuberant right inguinal hernia was managed surgically. The blinded study team assessed the maternal and fetal complications as unrelated to immunization.
3. A 19-year-old woman was found to have a spontaneous abortion 9 weeks into her third pregnancy and 9 weeks after her first and only dose of PfSPZ Vaccine. The temporal relationship to immunization led the team to consider the event possibly related to the vaccine. This event is further discussed below under SAEs.

#### Serious adverse events.

In addition to the three pregnancy complications, seven additional SAEs were recorded during the study; all were deemed unrelated to vaccination with the exception of a solitary seizure in a 15-year-old male, which was considered possibly related to immunization and, like the lost pregnancy, led to a temporary halt in trial execution. This participant received three doses of 1.8 × 10^6^ PfSPZ of PfSPZ Vaccine. He had a history of headaches occurring once every 2 weeks for approximately 1 year but otherwise had been in excellent health. No significant AEs were reported after the first two doses, and he remained well during 2 hours of observation at the clinical center after his third dose. Subsequently, while at school 3½ hours after vaccination, he had a witnessed seizure of 5 minutes’ duration that was initially focal and progressed to generalized. He was subdued after the seizure and complained of a severe headache but returned quickly to his normal level of alertness. There was no history of head trauma, fever, or other evidence of infection and no known history of seizure disorder. Neurological examination, laboratory tests, noncontrast computed tomography of the head, and magnetic resonance imaging of the brain after the seizure were normal. The initial electroencephalogram (EEG) showed frontal slow and paroxysmal abnormalities; a sleep-deprived EEG demonstrated findings consistent with a bifrontal focal or generalized epileptic seizure. Review by a specialist in epilepsy (coauthor O. D.) concluded that the most likely diagnosis was an idiopathic or genetic generalized epilepsy and suggested the potential role of the vaccine in this case was uncertain; however, the vaccine may have triggered a nonspecific immune response (e.g., cytokines) that lowered the seizure threshold. The participant completed the trial, with a normal EEG 168 days after the final immunization and no report of additional seizures 12 months after immunization.

The remaining six SAEs were all considered unrelated to immunization: acute low back pain in a 44-year-old, a fall with tongue laceration in an 11-month-old, gastroenteritis in a 9-month-old, abdominal pain after blunt trauma in a 13-year-old, cryptogenic pneumonia in a 2½-year-old, and malaria in a 2-year-old. Further details are available in the Supplemental Appendix.

#### Laboratory abnormalities.

The most frequent laboratory abnormality identified across all groups was mild neutropenia (Supplemental Table 8). Neutropenia was more frequent in the adult and adolescent groups, but the number of volunteers experiencing neutropenia was not statistically different between the vaccine recipients and controls in each group (*P* > 0.37 for all comparisons, Fisher’s exact test, two-tailed). Other commonly observed laboratory abnormalities included mild elevation of eosinophils (more frequent in NS control participants), mild decreases in hemoglobin, and mild elevations of aspartate aminotransferase (AST) and alanine transaminase (ALT). The frequencies of these abnormalities did not differ between the combined vaccine groups and control groups (*P* = 0.09, *P* = 1.00, *P* = 0.76, and *P* = 0.32, respectively, Fisher’s exact test, two-tailed). Grade 3 laboratory abnormalities included elevations in AST and ALT in one NS control and AST elevation (2), ALT elevation (1), thrombocytopenia (2), and lymphopenia (1) in four vaccinees. All grade 3 laboratory abnormalities resolved without sequelae. Only the episode of lymphopenia, which occurred 1 day after the second vaccine dose, was considered possibly related to the vaccine (Supplemental Table 9).

#### Plasmodium *parasitemia during and after immunizations.*

One child, a 2-year-old, developed symptomatic *P. falciparum* infection 4 weeks after the third immunization with PfSPZ Vaccine. No asymptomatic infections were detected in children. Seven adult participants (five vaccinees, two NS controls) were retrospectively determined by qPCR to have asymptomatic infections during the immunization period—one with *P. falciparum*, three with *Plasmodium malariae*, two with *P. falciparum* and *P. malariae*, and one with *P. falciparum* and *Plasmodium ovale* (Supplemental Table 10). One additional adult (41-year-old) vaccinee had asymptomatic *P. falciparum* infection detected 112 days after the third dose. This prevalence of asymptomatic infection among the adult study participants (19%) was comparable to the prevalence in healthy blood donors (29.5%) residing in the same region of Equatorial Guinea using the same molecular methods.^23^

### IgG antibodies to PfCSP.

**Figure 2.**
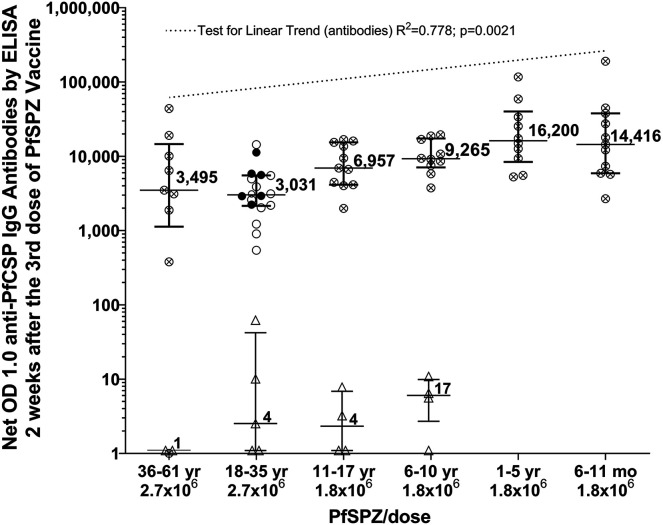
Net OD 1.0 IgG antibodies to PfCSP by ELISA 14 days after the third immunization with PfSPZ Vaccine or normal saline, by group. Horizontal arms represent medians and are bracketed by the interquartile range. Each point represents the results for a unique participant. Filled circles (⬤) represent participants not infected after CHMI; open circles (○) represent participants who were infected after CHMI (ages 18–35 years only).^14^ Crossed circles (⊗) represent participants who did not undergo CHMI, and open triangles (Δ) represent participants who received placebo. Net OD antibody levels were significantly different between vaccinees and placebo recipients in 18–35-year-olds (*P* < 0.001), 11–17-year-olds (*P* = 0.002), and 6–10-year-olds (*P* = 0.002) but not 36–61-year-olds (*P* = 0.11, Mann-Whitney two-sided test). No sera were available from controls in the 1–5-year-old and 6–11-month-old age groups. CHMI = controlled human malaria infection; PfCSP = *Plasmodium falciparum* (Pf) circumsporozoite protein; PfSPZ = *Plasmodium falciparum* sporozoites.

Serum antibody responses to PfCSP by ELISA for individual participants 2 weeks after the third immunization and ratios of post-immunization (post) to pre-immunization (pre) values are presented in Figure 2 (net OD 1.0), Supplemental Figure 1 (ratio OD 1.0), and Supplemental Table 11. Seroconversions (net OD 1.0 ≥ 50 and OD 1.0 ratio ≥ 3.0) occurred in 100% of the vaccinees in all age groups except 36–61-year-olds and none of the NS controls (*P* ≤ 0.002 in each of four age group comparisons between vaccinees and placebo recipients, Bernard’s two-sided test). In 36–61-year-olds, 78% of vaccinees (7/9) and 0% of controls (0/2) seroconverted (*P* = 0.08, Bernard’s two-sided test).

**Figure 3.**
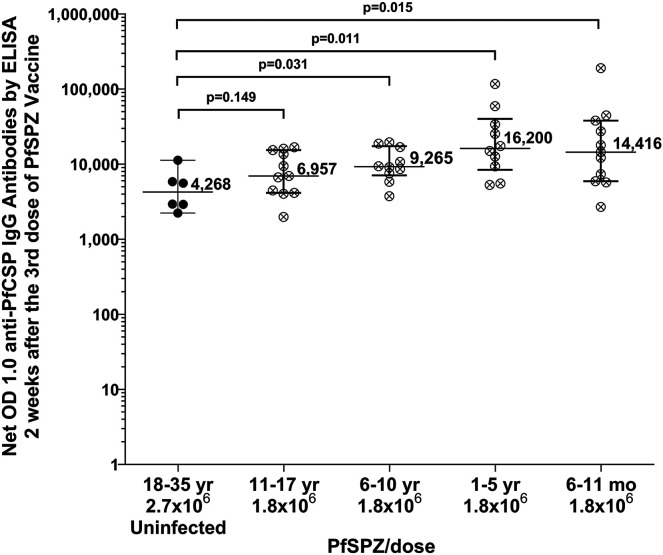
Comparison of Net OD 1.0 anti–PfCSP antibody levels in infants and children with levels in 18–35-year-old adults protected against *Plasmodium falciparum* parasitemia after CHMI. Horizontal arms represent medians and are bracketed by the interquartile range. Each point represents the results for a unique participant. Filled circles (⬤) represent participants not infected after CHMI; crossed circles (⊗) represent participants who did not undergo CHMI. Median antibody levels were significantly higher than the median for protected adults for children ages 6–11 months and 1–5 and 6–10 years. CHMI = controlled human malaria infection; PfCSP = *Plasmodium falciparum* circumsporozoite protein; PfSPZ = *Plasmodium falciparum* sporozoites.

To compare antibody responses to PfCSP between age groups, the ANOVA on rank test was performed separately for net OD 1.0 and OD 1.0 ratio (fold-change from baseline). The differences in mean antibody response levels between the groups for both net OD 1.0 and OD 1.0 ratio 2 weeks after third vaccination were highly significant (*P* < 0.0001, Kruskal-Wallis test). Participant antibody responses were significantly higher in the younger age groups than in adults, with a linear trend (net OD 1.0 *r*^2^ = 0.778; *P* = 0.0021 and OD 1.0 ratio *r*^2^ = 0.764; *P* = 0.0136) toward a higher antibody response after vaccination in each progressively younger age group (Figure 2, Supplemental Figure 1, and Supplemental Table 11). Median antibody responses in the three youngest age groups (6–11-month-olds, 1–5-year-olds, and 6–10-year-olds) were all significantly higher than the median antibody levels for 18–35-year-olds who did not develop parasitemia after CHMI (Figure 3). No difference was seen in median antibody levels between 36–61-year-olds and 18–35-year-olds (median OD 1.0 of 3,495 versus 3,031; *P* = 0.63). Interestingly, however, net OD 1.0 IgG levels correlated positively with age greater than 18 years (*r*^2^ = 0.218, *P* = 0.014).

Using age 11 years as an approximation to distinguish the effects of pre- and postpubertal differences between male and female participants, no difference was detected in median antibody levels between male (OD 1.0 of 13,518, *N* = 16) and female (OD 1.0 of 10,728, *N* = 15) participants < 11 years of age (*P* = 0.65, Mann-Whitney test). Likewise, no differences were detected in median antibody levels between male (OD 1.0 of 4,028, *N* = 29) and female participants (OD 1.0 of 5,599, *N* = 9) > 11 years of age (*P* = 0.66).

## DISCUSSION

This age escalation/de-escalation trial performed in malaria-exposed adults, children, and infants on Bioko Island, Equatorial Guinea, is the second trial to assess the safety, tolerability, and immunogenicity of PfSPZ Vaccine in infants, children, and adults and the first trial in older adults. To address vaccine safety, NS was chosen as a rigorous placebo control that can be safely administered by DVI. The frequency of local or systemic AEs was not significantly different between PfSPZ Vaccine and NS in all age groups combined or in each age group assessed individually (*P* > 0.27, Fisher’s exact test, two-tailed), nor were there any differences among age groups. This is the same finding as in a similar age de-escalation trial performed in malaria-exposed Tanzanian adults, children, and infants.^13^ In this Equatorial Guinea trial, headache was seen more frequently in vaccinees in each age group in which it was assessed (ages 6–10, 11–17, 18–35, and 36–61 years), but this difference was not statistically significant for any age group or when all four age groups were combined (*P* = 0.17). There was no trend toward increased frequency of headache with subsequent doses. Although frequently reported in both vaccine and control participants, a significant increase in headache has not been seen in any randomized controlled trial with PfSPZ Vaccine.^10^^–^^15^^,^^18^^,^^19^^,^^25^^–^^27^

As in other studies of PfSPZ Vaccine, DVI was well tolerated as a procedure. All participants in the vaccine and NS arms were successfully injected, requiring only a single needle stick in 91%. Success with the first attempt was lower in the 1–5-year-old (85%) and 6–11-month-old (80%) age groups than it was in 6–61-year-olds (95%), similar to what was previously reported in Tanzania (87% in 1–5-year-olds and 63% in 6–12-month-olds^13^) and Kenya (85% in 1–5-year-olds and 82% in 5–12-month-olds).^16^ Personnel administering vaccine or placebo by DVI in all three trials were new to the procedure, qualified by independently performing one successful DVI in an adult after training. In a concurrent trial in Kenya limited to infants aged 5–12 months at enrollment and relying upon the most skilled injectors, DVI success on the first attempt was 92%.^28^

Pain was subjectively rated by participants as mild or none in 93% of the injections in participants aged 6 years and above (Supplemental Table 6). Overall, acceptability of the DVI procedure was high, with no study withdrawals attributed to the procedure. In a previous analysis focusing on the acceptability of DVI in a study population in western Kenya, mothers found the procedure acceptable, with several reporting that their children had less pain and fewer side effects in comparison to those experienced after routine intramuscular immunizations.^29^ Good tolerability will facilitate good compliance should PfSPZ Vaccines be deployed in the future for mass vaccination programs.

There were two possibly related SAEs in the clinical trial. One was a solitary generalized seizure in a 15-year-old boy with no history of seizures, who was described as previously healthy. Seizures after immunization are reported with most licensed vaccines but are generally regarded as infrequent and typically associated with fever, although published trials frequently combine both types (with/without fever), blurring the distinction.^30^ In the initial report from the phase 3 RTS,S/AS01 trial, seizures were reported in 266 (211 febrile, 55 nonfebrile) of 5,949 participants receiving RTS,S (4.5%) and 143 (106 febrile, 37 nonfebrile) of 2,974 receiving the comparator rabies vaccine (4.8%).^31^ How vaccinations trigger nonfebrile seizures remains unknown, although there is increasing evidence that vaccines in general may serve as a trigger for seizures and the underlying etiology for the seizure is tied to a genetic predisposition.^32^^,^^33^ At the time of this event, there had been no other reports of seizure in association with immunization in any completed or ongoing trials of PfSPZ Vaccine. Subsequently, febrile and nonfebrile seizures have been reported in infants and children 5–16 months of age in western Kenya participating in PfSPZ Vaccine trials at a comparable frequency between participants receiving vaccine (19 of 364 participants, 5.2%) or NS (5 of 137 participants, 3.6%; *P* = 0.64, Fisher’s Exact test, two-tailed).^16^^,^^17^ These data are consistent with an absence of increased risk of seizures specific to PfSPZ Vaccine.

The second SAE was a spontaneous abortion at 9 weeks of pregnancy in a 19-year-old woman. By history, her last menstrual period was approximately 2 weeks prior to her first dose of PfSPZ Vaccine, implying conception occurred concurrently with administration of her first dose of PfSPZ Vaccine. Prior to this event, only one pregnancy had occurred in any trials of PfSPZ Vaccine (unpublished data); this participant had a positive urine pregnancy test 43 days after her fourth (final) immunization and 28 days after undergoing CHMI. This pregnancy was electively terminated 6 days later. Two additional pregnancies occurred during this trial, one of which produced a healthy child at 37.5 weeks. The second pregnancy was associated with complications determined by blinded study staff to be unrelated to immunization. The cumulative probability of miscarriage through 28 weeks’ gestation has been calculated at 18.9% in western Kenya,^34^ with 75% of miscarriages occurring by 18 weeks. Similar rates have been observed on the Thailand–Burma border (19%).^35^ In the absence of data and little precedent for PfSPZ Vaccine administration and pregnancy and with a substantial baseline risk for miscarriage, it is impossible to conclude that there is any association between immunization and spontaneous abortion or adverse fetal outcomes. We are actively further assessing the safety of PfSPZ Vaccine in women of childbearing potential in Mali and their offspring (https://clinicaltrials.gov/show/NCT03989102) as a first step toward accruing data on PfSPZ Vaccine safety in women who subsequently become pregnant.^20^

Compared with the 18–35-year-old group, antibody responses to PfSPZ Vaccine correlated inversely with age in the younger age groups, with maximum antibody levels in the 1–5-year-old age group, despite a higher vaccine dose administered to adults. Similar findings have been reported in Tanzania,^13^ with three doses of 9.0 × 10^5^ PfSPZ administered at 8-week intervals to participants age 6–11 months, 1–5 years, 6–10 years, 11–17 years, and 18–35 years (same age groups as in this study except for the older adults). All studies of PfSPZ Vaccine in African adults have shown diminished antibody responses to PfSPZ Vaccine^10^^–^^15^^,^^18^^,^^26^ compared with malaria-naive adults,^5^^–^^9^ supporting the hypothesis that prior chronic malaria infections lead to immune dysregulation, and one manifestation is reduced antibody responses. Assuming malaria exposure increases with age, antibody responses would be expected to show an inverse correlation with age as demonstrated in both the prior Tanzanian and current Equatorial Guinea studies. It appears that once adulthood is reached, there is no further decline in antibodies to PfCSP, as there was no difference in PfCSP antibody levels between 18–35-year-olds and 36–61-year-olds. In fact, linear regression analysis based on age showed a modest, but significant increase with age in 18–61-year-olds. The significance of this is unclear, and this observation awaits confirmation in larger clinical trials.

Antibody levels were higher in males than in females in children < 11 years of age and higher in females than males in participants ≥ 11 years of age, but neither difference was statistically significant. The latter finding is consistent with a meta-analysis of 11 previous clinical trials showing higher antibody responses in postpubertal females than males.^36^ The significance of the finding is unclear, however, as women did not experience improved VE compared with men in the studies included in the meta-analysis.^36^

Field trials with PfSPZ Vaccine in Mali and Burkina Faso, with data from men and women combined, have demonstrated a correlation between PfCSP antibody levels after immunization and protection from naturally transmitted *P. falciparum* infection.^18^^,^^19^ In all four younger age groups in the current study, median anti-PfCSP antibody levels were significantly higher than those in protected adults. These observations imply that PfSPZ Vaccine may show significant VE in younger age groups when tested in the field.

Antibody levels likely contribute to early VE by the neutralization of SPZ but, more importantly, may serve as a marker for protection that is mechanistically tied to cellular immune responses. A relationship between antibody levels and cell-mediate protection could result from Fc-mediated interactions, for example. Cellular immunity is thought to underpin the long-lasting immunity to naturally transmitted *P. falciparum* malaria seen in adults immunized with PfSPZ Vaccine.^10^^,^^18^^,^^36^

Notably, field protection has been achieved only in studies where trial participants were cleared of parasitemia prior to first immunization to eliminate the immunosuppressive properties of preexisting parasitemia. To realize the potential benefit of vaccination implied by the robust antibody responses of infants and children compared with adults in the current study, clearing parasitemia prior to immunization will likely be required. Only one study of PfSPZ Vaccine in a pediatric population has been published to date.^17^ In this study, conducted in Kenyan infants, clearance was not performed prior to immunization. Vaccine efficacy was seen at 3 months, likely explained by robust antibody responses, but waned thereafter, and no peripheral cellular immune responses to PfSPZ could be measured in any vaccinees in this study. Although immaturity of the infant immune system may have contributed, we suspect that failure to clear parasitemia was the major reason for poor VE.

Limitations of this study include the relatively small sample sizes, making it difficult to identify potential small differences in the rates of AEs between vaccinees and controls. Additional results, however, from a similarly designed trial in malaria-exposed Tanzanian infants, children, and adults^13^ and a trial in 501 Kenyan infants and children also showed good safety and tolerability,^16^^,^^17^ setting the stage for the conduct of larger studies.

There were no significant differences in AEs between vaccinated participants and controls. There was no indication of an increased frequency of AEs in young children and infants compared with older children or adults. Regarding immunogenicity, the trial showed the same inverse relationship between age and antibody levels as shown in a prior study in Tanzania, with levels in all pediatric age groups equal to or exceeding levels measured in adults protected after CHMI. These findings support the ongoing development of PfSPZ Vaccine to prevent *P. falciparum* malaria in children as well as adults.

## Supplemental Material


Supplemental materials
