# Supplementary material for: Safety and Immunogenicity of Radiation-Attenuated PfSPZ Vaccine in Equatoguinean Infants, Children, and Adults
Source: Am J Trop Med Hyg. 2023 May 9;109(1):138–46. doi: 10.4269/ajtmh.22-0773 (PMC10324022; doi:10.4269/ajtmh.22-0773)
Supplement: Supplementary file 1 [file tpmd220773.SD1.pdf]

Safety and immunogenicity of radiation-attenuated PfSPZ Vaccine in Equatoguinean infants, children and adults

SUPPLEMENTAL APPENDIX

<u>Reports of Serious Adverse Events</u>: The following narrative details each SAE reported during the course of the trial. A total of 10 SAEs were reported for this trial including 3 involving pregnancies that occurred during the course of the study. Two of the 10 SAEs were considered possibly related to vaccine – a spontaneous abortion at 9 weeks in a 19-year-old woman with an estimated date of conception coinciding with her first dose of $2.7x10^6$ PfSPZ of PfSPZ Vaccine, and a solitary, generalized tonic-clonic seizure in a 15-year-old male 3.5 hours after his 3rd dose of $1.8x10^6$ PfSPZ of PfSPZ Vaccine. These two SAEs are described first, followed by the eight unlikely related or unrelated SAEs. All SAEs were followed to a point of clinical stability or resolution. After the descriptions of the 10 SAE's, an additional event is described that involved an elective hospitalization to repair an umbilical hernia.

1. <u>Participant ID: E21A331 (PfSPZ Vaccine) – Spontaneous Abortion</u>

This 19-year-old woman, G3P2Ab0, was positive on a urine pregnancy test on 4 January 2017. At her screening visit on 22 October 2016, she had a normal physical examination and no significant past medical history. Screening hematology, biochemistry, urine and stool exams were unremarkable, and her hepatitis B, hepatitis C, HIV and urine pregnancy tests were negative. She had a menstrual period on 13 November 2016, and a second negative urine pregnancy test on 1 December 2016, at which time she was started on oral contraceptives and received her first dose of PfSPZ Vaccine. Since the first day of her last menstrual period was more than 7 days prior to the initiation of birth control pills, she was advised to use condoms in addition to the oral contraceptives for the first 30 days, according to WHO and CDC guidelines, and a supply of condoms was provided. She did not report any adverse events during 2-hour observation or 7-day surveillance period post study product administration. Her 28 days following vaccination were unremarkable. The volunteer notified the study team on 4 January that her menstrual period had not occurred in mid-December as expected. She was brought to the clinic and a urine pregnancy test was positive. Given that her LMP was 13 November, conception likely occurred in the same time frame as her first dose of PfSPZ Vaccine, which had been administered on 1 December.

According to the participant, she and her partner did not use condoms as had been recommended, but she reports having taken oral contraceptives as directed. She had no adverse events during follow-up visits and specifically denied vaginal bleeding, abdominal pain, nausea or other symptoms. She had had two previous full-term pregnancies; her first delivery was by cesarean section (indication unknown) two years earlier at the Malabo Regional Hospital, and her second delivery was vaginal at the Baney District Hospital seven months earlier. She stated that both of her sons weighed between 3 and 3.2 kg at birth and remained healthy. The participant initially stated that she did not wish to continue her pregnancy, and requested assistance from the study clinicians in obtaining an elective abortion, but this could not be provided because abortion is illegal in Equatorial Guinea.

On 13 January 2017 the participant was contacted by phone and reversed her concerns about

the pregnancy, requesting antenatal care at La Paz Medical Center (a well-equipped, local private hospital that is also the hospital used to care for study participants). On 17 January 2017 she was evaluated at La Paz by a maternal fetal medicine specialist. She was asymptomatic at 9 2/7 weeks gestational age by LMP, but a transvaginal ultrasound showed a 6-week sized embryo without cardiac activity. A follow-up transvaginal ultrasound by the same specialist was scheduled for one week later to confirm the status of the embryo. On 24 January 2017 the volunteer remained asymptomatic at 10 2/7 weeks by LMP, and the repeat transvaginal ultrasound showed no cardiac activity and no growth; hence the diagnosis of pregnancy loss was confirmed. She did not report any abdominal pain or vaginal bleeding. Vital signs were normal; blood pressure 102/70 mmHg, pulse rate 62/min. The participant was prescribed misoprostol 400 mg PO and 400 mg intravaginally on 24 January 2017. She reported passage of a significant amount of tissue and blood on 25 January 2017; by the following day she had no further vaginal bleeding and was pain free. Subsequent transvaginal ultrasound revealed no evidence of retained products of conception in the uterus. She was discharged from care by the gynecologist on 26 January 2017. She was seen again at the Baney Research Center on 7 February 2017 and denied any abdominal pain or vaginal bleeding.

Due to the fact that conception and the first dose of PfSPZ Vaccine occurred in the same time frame, the loss of this embryo was deemed a possibly related SAE by the study team. Per the protocol, the study was placed on a safety hold (possibly related SAE). An ad hoc data and safety monitoring board (DSMB) committee meeting was convened on 26 January 2017 to review the case and to make an assessment regarding the safety hold. The DSMB agreed with withholding further immunizations in the research participant who became pregnant. Regarding the rest of the research participants, the DSMB recommended resuming immunizations with enhanced measures to assure adherence to the pregnancy prevention guidelines present in the protocol for women of child-bearing potential, adding the requirements to be on adequate birth control for two weeks prior to immunization and to repeat pregnancy counseling at every study visit, the latter to be documented in the participant's chart. The DSMB did not recommend changes in the study consent or assent forms for women of childbearing potential, which clearly warn volunteers about the potential dangers of pregnancy and the importance of adhering to birth control guidelines. The study team developed a new SOP to enact the enhanced measures to assure adherence to the pregnancy prevention guidelines in the protocol.

The four oversight committees (the National Ethics Committee of Equatorial Guinea, the Ifakara Health Institute IRB, the Ethics Committee of Northwestern and Central Switzerland and the MaGil IRB, USA) and the US FDA were also notified. No additional recommendations were provided beyond those of the DSMB. All study sites using PfSPZ Vaccine were notified of this event and the importance of avoiding pregnancy was emphasized.

2. Participant ID: E203347 (PfSPZ Vaccine) – Solitary Seizure

This 15-year-old male had a history of new onset diffuse headaches approximately every two weeks for the preceding year but otherwise had been in excellent health, and was reported to be a student in good standing at his school. He received three doses of PfSPZ Vaccine ($1.8x10^6$ PfSPZ administered by DVI on 07 December 2016, 24 February 2017, and 19 April 2017). All three doses appeared to be well tolerated without noteworthy adverse events during follow-up. This included two hours of observation at the clinical center after his 3rd dose on 19 April 2017, where he ate lunch and felt normal after the injection. The participant then went to

school where a seizure occurred, witnessed by classmates. It began with trembling of the right hand while the participant was awake and aware that he could not control the movement, followed by spontaneous lifting of the arm. He remembered falling towards a friend and then lost consciousness until the seizure ended approximately five minutes later. The seizure was characterized by eye rolling, hypersalivation, and tongue biting, but not by incontinence. He awoke with a transient headache. He was subdued after the seizure, but returned quickly to his normal level of alertness and has remained well since the event. There was no history of head trauma, fever or other evidence of infection. There was no known history (or family history) of seizure disorder. Neurological examination and funduscopic examination after the seizure were normal. All laboratory tests were normal. A non-contrast CT of the head and an MRI of the brain were confirmed as normal by a neuroradiologist at New York University (NYU) Medical Center. The initial EEG was read by the neurologist in Equatorial Guinea as showing frontal slow and paroxysmal abnormalities supporting a partial epileptic seizure of frontal type; a second sleep deprived EEG was abnormal, with the findings consistent with a frontal seizure focus. Review of the EEGs by a specialist in epilepsy at NYU concluded "Epileptiform activity is present that supports the diagnosis of a generalized epilepsy or tendency for generalized epilepsy…Given the clinical, imaging and EEG features in this case, the most likely diagnosis is an idiopathic or genetic generalized epilepsy. The potential role of the vaccine is uncertain. If there is a role, it is most likely that the vaccine triggered a non-specific immune response (e.g., cytokines) that lowered the seizure threshold in an individual with a genetic (or previously acquired) tendency towards seizures and/or epilepsy."

The seizure was deemed a possibly related SAE by the study team. The study was placed on hold (possibly-related SAE) 20 April 2017. In an ad hoc data and safety monitoring board (DSMB) meeting 21 April 2017 the DSMB agreed with withholding further immunizations pending further evaluation of the participant, and recommended informing all IRBs, regulatory authorities and other sites testing PfSPZ Vaccine or other Sanaria PfSPZ-based products that the SAE had occurred. Sanaria informed all regulatory authorities including the US FDA and principal investigators at other sites of this event, as well as members of all SMCs and DSMBs convened by Sanaria. At a second meeting 8 May 2017, the DSMB reviewed all reports pertaining to the seizure and the collective experience with PfSPZ Vaccine in all adult and pediatric trials. The DSMB recommended the trial could be resumed, concurring with Sanaria's plans to add specific screening questions for seizure history, review the seizure history for all enrolled participants, add history of non-febrile seizures as an exclusion criterion by protocol amendment and specifically inform the volunteer and his family, all IRBs with oversight of the EGSPZV2 trial, all PIs of trials using PfSPZ-based products, all SMCs/DSMBs that advise Sanaria on clinical trials and all sponsors that cross-reference Sanaria INDs for the INDs of their specific trials.

Because the study participant received all three immunizations with PfSPZ Vaccine, there was no perceived need to withdraw him from the study, as all remaining study visits consisted of safety follow-up with the exception of minor blood draws for research laboratory tests. At completion of the study, 168 days after the 3rd immunization, the participant appeared in good health with normal hematology and biochemistry. He was not on anticonvulsants and there had been no report of further seizures. A follow up EEG performed 1 November 2017 was read as normal.

3. <u>Participant ID: E202351 (PfSPZ Vaccine) – Acute Low Back Pain</u>

This 44-year-old male participant had an acute onset of severe (grade 3) low back pain on 8 December 2017, 34 hours after receiving his first immunization with $1.8x10^6$ PfSPZ of PfSPZ Vaccine. He presented to the La Paz Medical Center emergency department via ambulance just after midnight on 9 December 2017. He did not report any history of injury, radiation of the pain to the lower extremities, blood in urine or painful micturition. This was the first time the volunteer had experienced this type of pain. Aside from a blood pressure of 142/98 at screening visit 1 which had normalized on the day of vaccination there no noteworthy past medical history.

On examination, the emergency department physician at La Paz Medical Center noted severe tenderness at the left lower back. Blood pressure was elevated to 138/98. All other vital signs were normal and systematic examination revealed no other abnormalities. A lumbosacral x-ray showed no abnormalities and the full blood count was normal. The volunteer had a slightly raised blood urea level of 22 mg/ml (normal 7-19) but the creatinine level and urinalysis were normal. He was given metamizole (an analgesic/antispasmodic) 1mg plus diazepam 5mg intravenously once. The severity of the pain subsided rapidly and all pain resolved completely within a few hours. Given the initial severity of the pain and the late hour, hospital admission for the purpose of observation was done. The pain resolved without any sequelae and the volunteer was discharged within 24 hours. Given the temporal proximity to immunization and the lack of an alternative etiology, this SAE was initially deemed to be possibly related to the IP.

Follow-up 14 December 2016 revealed no recurrence of back pain. Blood pressure was 123/93. Lifestyle modification was advised. On 19 December 2016 the blood pressure was 126/85 and the blood urea level remained elevated at 23.9. On 4 January 2017 the blood pressure was 137/93 and the urea level was normal at 18.8. The back pain did not recur and the participant received his second and 3rd immunizations as planned.

Subsequent to the trip to the emergency room and brief hospitalization, this study participant voluntarily admitted that the severity of the back pain was purposely exaggerated in order to prove to an acquaintance that his participation in the trial had empowered him to visit the La Paz Medical Center emergency department at will. The event remains technically classified as an SAE (there was a hospitalization) but it was reclassified as unrelated to vaccination.

4. <u>Participant ID: E26A532 (PfSPZ Vaccine) – Fall with Tongue Laceration</u>

This 11-month-old boy was in good health without any adverse events recorded following a first immunization with $9x10^5$ PfSPZ of PfSPZ Vaccine on 22 March 2017. Thirty-one days after immunization, the child fell from a standing position onto a concrete floor and was found afterwards to be bleeding transiently from the mouth. An hour later the child fell again, and there was more bleeding from the mouth and prolonged crying. The child was examined at a local hospital and sent home. Bleeding reoccurred and the child was taking to La Paz Medical Center where a tongue laceration was identified and the child was admitted for observation. Complete blood count was normal but coagulation tests were slightly elevated (prothrombin time 14.3 seconds, normal range 11-14 seconds; partial thromboplastin time 36.2 seconds, normal range 25-35 seconds; international normalized ratio (INR) 1.32, normal range 0.8 to 1.2). Bleeding did not reoccur and the child was discharged on the day after admission without further complications.

The child did not appear to sustain a head injury and did not lose consciousness after the falls. The mother was with the child the first time he fell and reports no evidence of seizure activity. The mother was not with the child the second time he fell, but the child started crying and the mother came to his aid immediately, and according to the mother there was no evidence of seizure activity. The child had not been sick at the time of the injury. This SAE was considered not related to PfSPZ Vaccine administration.

5. Participant ID: E26B594 (PfSPZ Vaccine) – Gastroenteritis

This 9-month-old male was well until 10 September 2017 when he was admitted to the local hospital with sudden onset severe watery diarrhea, vomiting and dehydration. Diarrhea was non-bloody. There was an antecedent history of nonproductive cough but no fever. The child had been treated for an abscess on 16 August with amoxicillin and ibuprofen in the clinic. Admission CBC and biochemistry tests were normal except for an elevated BUN with a normal creatinine, consistent with intravascular volume depletion. The child was treated with IV hydration and amoxicillin-clavulanate for a respiratory infection, improved and was discharged 14 September 2017. He was well at clinic follow up 19 September 2017.

The prior vaccine dose (dose #1, $1.8x10^6$ PfSPZ) was administered 1 August 2017. This SAE was not considered related to PfSPZ Vaccine administration.

6. Participant ID: E205533 (PfSPZ Vaccine) – Malaria

This 2-year-old boy was hospitalized 22 September 2017 with fever and vomiting. He had a temperature of 39.5°C; a blood smear for malaria was "4+" positive for *P. falciparum.* A repeat blood smear 23 September was positive with 1647 parasites/µL. He was treated with parenteral artesunate (34 mg at 0, 12, 24 and 48 hours) and cefuroxime; this was followed up in the clinic with artemether-lumefantrine (20/120 mg) twice daily for 3 days and oral cefuroxime x 3 days. Repeat blood smears 25 September and 6 October were negative. Recovery was uneventful and without sequelae. The discharge diagnoses were malaria and acute tonsillitis, with malaria as the cause of the SAE.

Of note, this participant was seen in the clinic 6 days previously, on 16 September 2017, with a diagnosis of acute gastroenteritis and was treated with oral rehydration therapy and paracetamol. There is no record of malaria smears from that time point. The previous immunization (dose #3, $1.8x10^6$ PfSPZ) was administered on 29 August 2017. This SAE was considered not related to immunization.

7. Participant ID: E203320 (PfSPZ Vaccine) – Hyperemesis Gravidarum

An 18-year-old woman noted onset of vomiting approximately 5 days prior to hospital admission (on 1 September 2017). Vomiting was initially 3 times/day and she felt weak. She reported feeling feverish the following day. Two days prior to admission she had a positive urine pregnancy test. Her vomiting and generalized weakness progressed, leading to her presentation to her local hospital. She did not report abdominal pain, diarrhea, vaginal discharge, dysuria or urinary frequency. Her hemoglobin was 12.4 gm/dL. Serology for HBV, HCV and HIV were negative. A Widal test was positive – on this basis ampicillin was recommended but she did not take this, and improved with IV fluids and vitamins.

This participant's 3rd vaccine dose ($1.8x10^6$ PfSPZ) had been administered on 19 April 2017. Her last menstrual period was 18 July 2017. The remainder of her pregnancy was uneventful and she delivered a healthy baby girl at 37 + 4 weeks. Her SAE was judged to be not related to vaccine.

8. Participant ID: E203455 (PfSPZ Vaccine) – High risk pregnancy/IUGR

This previously healthy 15-year-old, G1P0, was found to have a positive urine pregnancy test on 18 April 2017. She received 2 doses of $1.8x10^6$ PfSPZ of PfSPZ Vaccine on 8 December 2016 and 25 February 2017 without incident; the 3rd dose was not administered. She reported her last menstrual period was 29 March 2017; a follow-up ultrasound estimated the date of conception as 7 April 2017. She entered antenatal care, including Fansidar (sulfadoxine-pyrimethamine) for intermittent preventive treatment for malaria. The pregnancy progressed normally with normal fetal ultrasounds on 14 July and 1 September 2017. Ultrasound on 25 October 2017 revealed a fetus with an estimated weight of 1129 grams, equating to the 1st percentile for the estimated gestational age of 30+5 weeks. Intrauterine growth restriction (IUGR) was confirmed by Doppler ultrasound on 1 November 2017; no fetal malformations were noted. IM betamethasone was administered for fetal pulmonary maturation. A repeat ultrasound again revealed a fetus with an estimated weight of 1452 grams (0 percentile) with borderline oligohydramnios and increased placental resistance. At the same time the participant had mild facial and ankle edema, a blood pressure of 142/95 and 2+ proteinuria. She was admitted to the hospital and underwent cesarean section 17 November 2017 at 33+2 weeks; the infant weighed 1300 grams with Apgar scores of 8 and 9.

The volunteer had no further complications and recovered fully. The infant was initially hypoglycemic but responded to treatment; parenteral antibiotics were administered although no evidence of neonatal sepsis was found. The baby was discharged after 3 days and gained weight appropriately at home. A patent ductus arteriosus (PDA) was identified on echocardiogram on 4 December and treated with 2 days of ibuprofen; repeat echocardiograms dated 31 January and 30 April 2018 showed the PDA had decreased in size. Also noted were moderate tricuspid insufficiency and a patent foramen ovale. Cardiac chamber sizes and systolic function were normal. The child also had a protuberant right inguinal hernia and underwent bilateral inguinal herniorrhaphies under general anesthesia on 26 December 2017 without complication.

A pediatric cardiologist from the University of Maryland was asked to review this case and was provided all available reports, labs and cardiac ultrasounds. It was concluded that the cardiac abnormalities were sufficiently stable and likely to remain unchanged or perhaps improve over a course of years. No further interventions were recommended.

9. Participant ID: E205541 (Normal Saline) – Cryptogenic Pneumonia

This 2.5-year-old female participant received first and second immunizations as a normal saline control on 28 March 2017 and 4 July 2017. Prior to her third immunization, the participant presented to her local hospital 24 August 2017 with high grade fever (T 39.3°C), cough and difficulty breathing (respiratory rate 24/minute, oxygen saturation 99%). Her initial symptoms began approximately 2 weeks prior to admission and she was seen in the study clinic on 17 August 2017 with stable vital signs, a normal physical examination, a negative

malaria smear and a hemoglobin 9.6 gm/dL with a total WBC of 13,900 cells/µL (12.8% neutrophils). She was sent home on amoxicillin but acutely worsened on the day of admission. Her WBC was 16,000 cells/µL (54.3% neutrophils); chest radiograph showed diffuse left lung infiltrates. She was treated with intravenous ceftriaxone (5 days) and oral azithromycin (4 days) with improvement and she was discharged to home on oral amoxicillin-clavulanate (10 days) + azithromycin (1 day).

Fever persisted and the participant was re-admitted 31 August 2017 with temperature 39.4°C, HR 140/minute and respiratory rate 24/minute. Chest radiographs now showed consolidation in the left base and she was treated with 7 days parenteral cefotaxime. She again improved and was discharged 7 September on 10 days of amoxicillin-clavulanate. In the clinic on 11 September the participant had an oral temperature of 38.7°C and she was re-admitted to the hospital. All cultures were negative, HIV serology was negative, malaria smears were negative. Non-contrast chest CT showed dense left lower lobe consolidation with a small pleural effusion and left hilar adenopathy. She was treated with parenteral vancomycin (10 days) and imipenem-cilastatin (7 days) for suspected resistant community acquired pneumonia (CAP) and once again improved. Over the next 4 weeks the mother reported intermittent episodes of fever; in the clinic on 23 October 2017 temperature was 38.9°C and abnormal breath sounds in the left chest. Amoxicillin-clavulanate was restarted but on return to the clinic on 25 October 2017 temperature persisted at 39.4°C with a respiratory rate of 39/minute. Azithromycin was added without benefit and she was again admitted on 27 October 2017 for fever, cough and tachypnea. She was thought to have slowly resolving CAP, microctic anemia and a small PDA (noted on a previous echocardiogram). She was treated with piperacillin-tazobactam (1 day) followed by imipenem-cilastatin + parenteral sulfamethoxazole-trimethoprim for 21 days; she also completed 10 days of oral azithromycin and one week of prednisolone and bronchodilator therapy. She defervesced on 2 November and remained afebrile at discharge.

The child was readmitted 29 November 2017 with a history of fever. She had decreased breath sounds in the left lower 2/3 of her chest and alveolar infiltrates in the right lower lobe and the left base. Parenteral vancomycin and amikacin were initiated. The case was presented to the DSMB on 8 December 2017; the members agreed with the attending physician's recommendation for bronchoscopy and suggested CT angiography might also be indicated. On 18 December antibiotics were discontinued and the child was transferred to the Centre Hôspitalier Universitaire de Libreville (CHUL) in Gabon and underwent bronchoscopy on 20 December. Concentric stenosis of the left mainstem bronchus was seen. Aspirates for AFB and GeneXpert were negative. The chest CT from that hospitalization was reviewed by a pediatric radiologist at the University of Maryland and demonstrated persistent opacification with some cavities and bronchiectasis in the central left lower lung. At the most recent telephone call on 22 March 2018 the mother reported the child was in good health with no return of fever or cough. A final recommendation was made to pursue repeat chest radiograph in 6-12 months with a community physician. This SAE was considered unlikely related to vaccine.

10. <u>Participant ID: E203350 (PfSPZ Vaccine) – Abdominal Pain</u>

This is a 13-year-old boy who sustained blunt trauma to the abdomen after a fall while playing on 30 August 2017. His examination in the local emergency department revealed mild to moderate epigastric tenderness and a protruded, reducible umbilicus. An abdominal ultrasound was normal. He was managed conservatively at home with paracetamol and ibuprofen. On

return visits to the clinic 4 September and 6 September he still complained of pain; examinations consistently showed normal vital signs and mild epigastric tenderness. He was referred to a local surgeon; it was decided the pain might be related to the umbilical hernia and this was electively repaired on 15 September 2017. This participant had his 3rd immunization on 19 April 2017 ($1.8x10^6$ PfSPZ). Although originally reported as an SAE, this event was downgraded to unsolicited AE as the surgical repair was a planned procedure. This AE was judged not related to vaccine.

Inclusion Criteria

1. Healthy males and females, based on clinical and laboratory findings
2. Age 6 months to 65 years
3. Adults with a Body Mass Index (BMI) 18 to 30 Kg/m^2; or adolescents, children and infants with Z-score of the selected indicator ([weight-for-height], [(height and BMI) for age]) category within ±2SD.
4. Long-term (at least one year) or permanent residence in the Baney district or Malabo city
5. Agreement to release medical information and to inform the study doctor concerning contraindications for participation in the study
6. Willingness to be attended to by a study clinician and take all necessary medications prescribed during study period
7. Agreement to provide contact information of a third-party household member or close friend to study team
8. Agreement not to participate in another clinical trial during the study period
9. Agreement not to donate blood during the study period
10. Able and willing to complete the study visit schedule over the study follow up period, including the hospitalizations required for protocol compliance
11. Willingness to undergo HIV, hepatitis B (HBV) and hepatitis C (HCV) tests
12. Volunteer (participants 18 years of age and older) or the parent / guardian signing the informed consent (for participants <18 years of age) is able to demonstrate their understanding of the study by responding correctly to 10 out of 10 true/false statements (in a maximum of two attempts for those who failed to respond correctly to all true/false statements in the first attempt).
13. Signed written informed consent, in accordance with local practice, provided by adult volunteers, parents or legal representatives and relevant assent for children participants as applicable.
14. Free from malaria parasitemia by blood smear at enrollment and by PCR for group 1
15. Has not been treated with any antimalarial medication for at least two weeks prior to the first immunization.
16. Free from helminth infections (detected by microscopy) at enrollment.
17. Female volunteers aged 9 years and above must be non-pregnant (as demonstrated by a negative urine pregnancy test), and those aged 13 to 49 years provide consent/assent of their

willingness to take protocol-defined measures not to become pregnant during the study and safety follow-up period.

Exclusion Criteria

1. Previous receipt of an investigational malaria vaccine in the last 5 years
2. Participation in any other clinical study involving investigational medicinal products including investigational malaria drugs within 30 days prior to the onset of the study or during the study period
3. History of arrhythmias or prolonged QT-interval or other cardiac disease, or clinically significant abnormalities in electrocardiogram (ECG) at screening
4. Positive family history in a 1st or 2nd degree relative for cardiac disease at age <50 years old
5. A history of psychiatric disease
6. Suffering from any chronic illness including; diabetes mellitus, cancer or HIV/AIDS
7. Any confirmed or suspected immunosuppressive or immune-deficient condition, including asplenia
8. History of drug or alcohol abuse interfering with normal social function
9. The use of chronic immunosuppressive drugs or other immune modifying drugs within three months of study onset (inhaled and topical corticosteroids are allowed) and during the study period
10. Any clinically significant deviation from the normal range in biochemistry or hematology blood tests or in urine analysis
11. Positive HIV, hepatitis B virus or hepatitis C virus tests
12. Volunteers who are have risk factors for tuberculosis and/or signs and symptoms of tuberculosis (TB), plus a positive tuberculin skin test (TST).
13. Symptoms, physical signs and laboratory values suggestive of systemic disorders including renal, hepatic, blood, cardiovascular, pulmonary, skin, immunodeficiency, psychiatric, and other conditions which could interfere with the interpretation of the study results or compromise the health of the volunteers
14. Any medical, social condition, or occupational reason that, in the judgment of the investigator, is a contraindication to protocol participation or impairs the volunteer's ability to give informed consent, increases the risk to the volunteer because of participation in the study, affects the ability of the volunteer to participate in the study or impairs the quality, consistency or interpretation of the study data.
15. History of non-febrile seizures or atypical febrile seizures.

Figure S1: IgG Antibodies to PfCSP by ELISA, by Group, expressed as the ratio of the OD 1.0 antibody levels 14 days post 3rd immunization to pre-immunization. This graph compares only the study arms receiving PfSPZ Vaccine. Horizontal arms represent medians and are bracketed by the interquartile range. Each point represents the results for a unique participant. Filled circles (●) represent participants not infected after CHMI; open circles (○) represent participants who were infected after CHMI (Ages 18 to 35 years only) (1). Crossed circles (⊗) represent participants who did not undergo CHMI and open triangles (Δ) represent participants who received placebo. The ratio of O.D. antibody levels were significantly different between vaccinees and placebo recipients in 18-35-year-olds ($p<0.001$), 11-17-year-olds ($p=0.002$) and 6-10-year-olds ($p=0.002$) but not 36-61-year-olds ($p=0.11$, Mann-Whitney 2-sided test). No sera were available from controls in the 1-5-year-old and 6-11-month-old age groups.

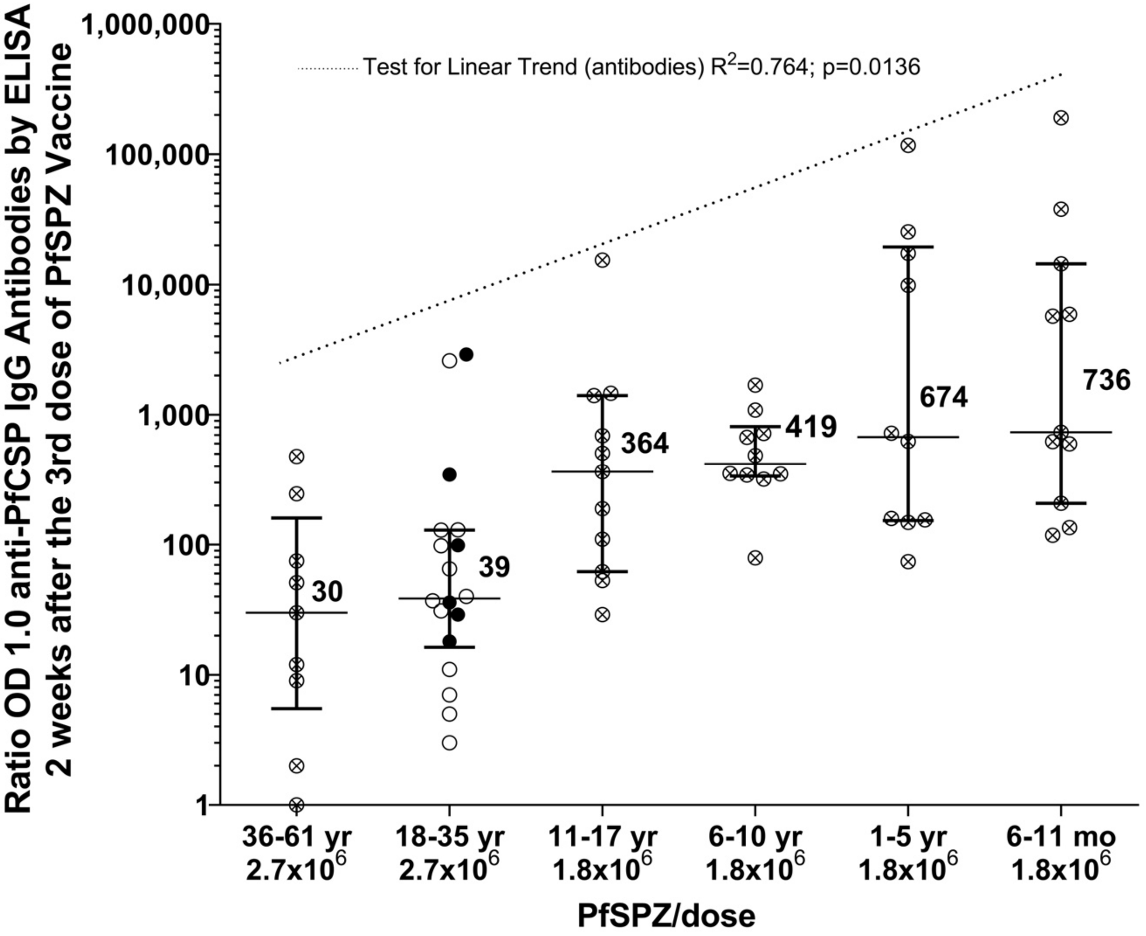

Figure S2: Comparison anti-PfCSP antibody levels in infants and children to levels in 18-35-year-old adults protected against Pf parasitemia after CHMI, expressed as the ratio of the OD 1.0 antibody levels 14 days post 3rd immunization to pre-immunization. Horizontal arms represent medians and are bracketed by the interquartile range. Each point represents the results for a unique participant. Filled circles (●) represent participants not infected after CHMI; crossed circles (⊗) represent participants who did not undergo CHMI. Median antibody levels were significantly higher than the median for protected adults for children ages 6-11 months and 1-5 years.

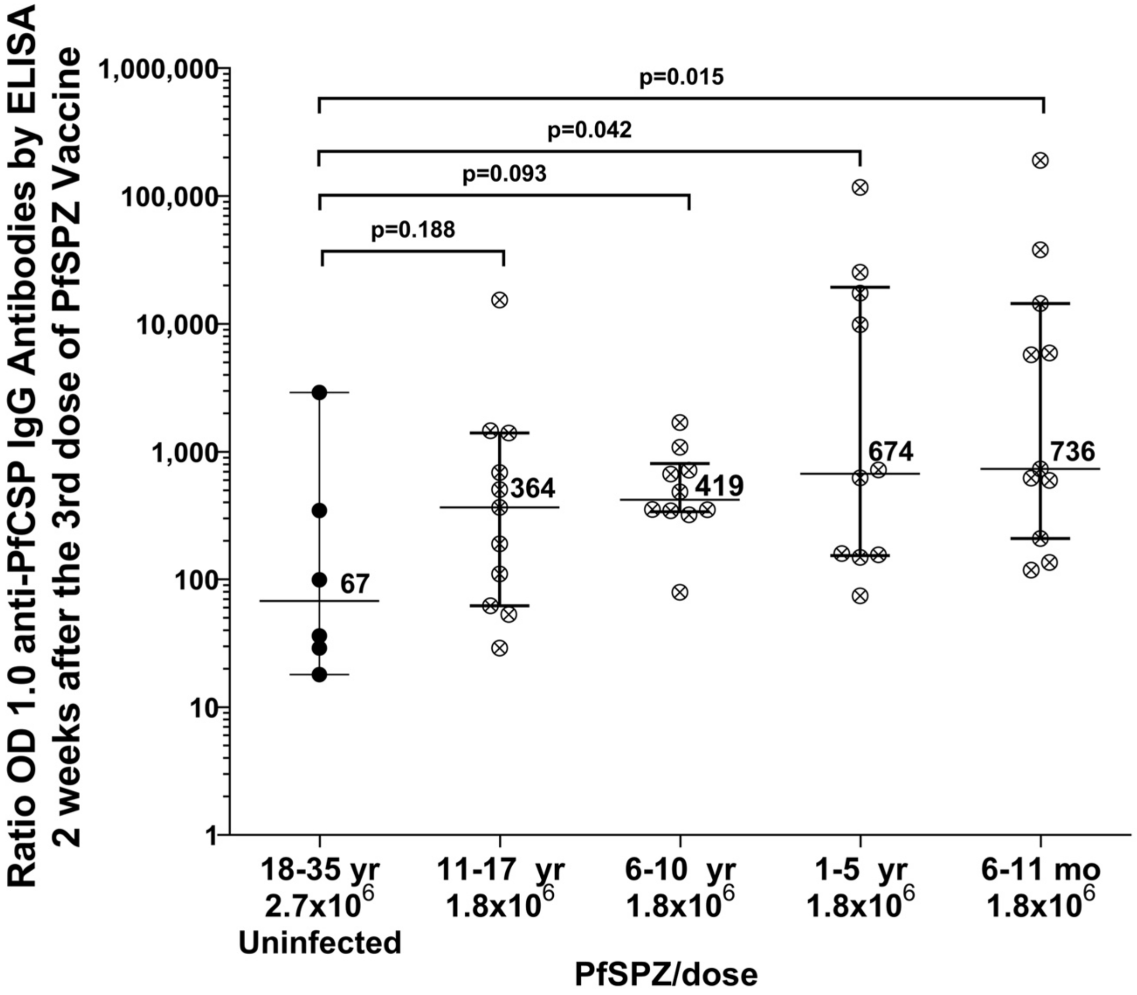

Table S1: Participant allocation to PfSPZ Vaccine (enrollment of each group was done sequentially)

Study Group	Age	Treatment Group	# Doses	# Volunteers Planned	# Volunteers Enrolled	Order of initiation*	Gap (weeks from prior group)
1	18-35 years	2.7×10^{6} PfSPZ Vaccine	3	20	20	1	not applicable
		Placebo	3	6	6		
2	36-61 years	2.7×10^{6} PfSPZ Vaccine	3	12	12	2	1
		Placebo	3	4	3		
3	11-17 years	1.8×10^{6} PfSPZ Vaccine	3	12	12		
		Placebo	3	4	4		
4	6-10 years	1.8×10^{6} PfSPZ Vaccine	3	12	12	3	6
		Placebo	3	4	4		
5	1-5 years	1.8×10^{6} PfSPZ Vaccine	3	12	11	4	9
		Placebo	3	4	4		
6A	6-11 months	9.0×10^{5} PfSPZ Vaccine	1	3	3		
6B	6-11 months	1.8×10^{6} PfSPZ Vaccine	3	12	12	5	1
		Placebo	3	4	3		
**Total**				**109**	**106**		

* Order of initiation staggered to assure safety prior to each age escalation, age de-escalation, or dose escalation.

Table S2: List of solicited adverse events with the grading system for severity*.

			Severity Grade	Description
Local Solicited AEs *(at injection site)*		• Pain<br>• Tenderness<br>• Pruritus	1	Daily activity minimally affected, with or without treatment
			2	Daily activity possible but only with treatment
			3	Daily activity not possible even with treatment
		• Erythema<br>• Swelling<br>• Induration<br>• Bruising/extravasated blood	1	2.5 – 5 cm
			2	5.1 – 10 cm
			3	>10 cm, necrosis or exfoliative dermatitis
Systemic Solicited (Core List-post Vaccination)		• Fever	1	38.0°C – 38.4°C
			2	38.5°C – 38.9°C
			3	>39.0°C
	Adults, adolescents and older children	• Allergic reaction *(rash, urticaria, pruritus, edema)*<br>• Headache<br>• Subjective Fever**<br>• Fatigue<br>• Malaise<br>• Chills<br>• Myalgia<br>• Arthralgia	1	Daily activity minimally affected, with or without treatment
			2	Daily activity possible but only with treatment
	Infants and younger children	• Allergic reaction *(rash, urticaria, pruritus, edema)*<br>• Subjective fever**<br>• Drowsiness<br>• Irritability/fussiness<br>• Inability/refusal to eat or drink	3	Daily activity not possible even with treatment

*AEs (solicited and unsolicited) were recorded and graded by physicians: mild (easily tolerated), moderate (interfere with normal activity), severe (prevents normal activity) or life threatening (Table S3). Axillary temperature was Grade 1 (38.0-38.4°C), Grade 2 (38.5–38.9°C) or Grade 3 (> 39.0°C). Hematological and biochemical abnormalities were assessed using standard clinical assays. All AEs were assessed for severity and relatedness to IP administration. AEs were classified as definitely related, probably related, possibly related, unlikely to be related, or not related. Definitely, probably, and possibly were classified as related to IP administration; unlikely to be related and not related were classified unrelated.
** Perceived by the volunteer and/or volunteer's guardian

Table S3: Locally derived lab normal ranges and toxicity grading scales.

NORMAL RANGES AND TOXICITY GRADING FOR CLINICAL LABORATORY PARAMETERS; HEALTHTY 6 MONTHS TO 10 YEARS								
**Biochemistry Parameters**	**Age Group**	**Unit**	**Normal Range**		**Mild (Grade 1)**	**Moderate (Grade 2)**	**Severe (Grade 3)**	**Potentially life Threatening (Grade 4)**
			**LLN**	**ULN**				
Alanine aminotransferase (ALAT)	3mo - 12mo	U/L	9	33	>33 - <66	66 - <99	99 - <264	> 264
	1y - 5y	U/L	10	28	>28 - <56	56 - <84	84 - <224	> 224
	6y - 10y	U/L	9	35	>35 - <70	70 - <105	105 - <280	> 280
Aspartate aminotransferase (ASAT)	3mo - 12mo	U/L	26	65	>65 - <130	130 - <195	195 - 520	> 520
	1y - 5y	U/L	27	55	>55 - <110	110 - <165	165 - 440	> 440
	6y - 10y	U/L	21	51	>51 - <102	102 - <153	153 - 408	> 408
Total Bilirubin (TBIL), normal liver enzymes	3mo - 12mo	mg/dL	0	1.11	0.12 – 1.66	1.67 – 2.21	2.22- 3.33	> 3.33
	1y - 5y	mg/dL	0	0.52	0.53 – 0.78	0.79 -1.04	1.05 – 1.58	> 1.58
	6y - 10y	mg/dL	0	0.64	0.65- 0.95	0.96 -1.27	1.28 – 1.93	> 1.93
Creatinine	3mo - 12mo	mg/dL	0.14	0.32	0.33 - 0.80	0.81 - 1.10	1.11 – 1.50	> 1.50
	1y - 2y	mg/dL	0.17	0.57	0.58 – 0.80	0.81 – 1.10	1.11 – 1.50	>1.50
	2y - 5y	mg/dL	0.17	0.57	0.58-1.0	1.01 – 1.60	1.61 – 2.0	> 2.0
	6y - 10y	mg/dL	0.27	0.55	0.56 – 1.0	1.01 – 1.60	1.61 – 2.0	>2.0

NORMAL RANGES AND TOXICITY GRADING FOR CLINICAL LABORATORY PARAMETERS; HEALTHY 6 MONTHS TO 10 YEARS

Hematology Parameters	Age Group	Unit	Normal Range		Mild (Grade 1)	Moderate (Grade 2)	Severe (Grade 3)	Potentially life Threatening (Grade 4)
			LLN	ULN				
HGB – Hemoglobin	6mo - 2y	g/d L	8.6	13.2	8-8.6	7-7.9	<7	Cardiac Failure secondary to anemia
	2y -5y	g/d L	8.6	13.2	8-8.6			
	6y - 10y	g/d L	9.6	14.1	8-9.6			
PLT – Platelets	6mo -5y	10^3/µL	91	491	74.999 - 90.999	50 - 75	25 - 49.999	<25
	6y - 10y	10^3/µL	91	456				
WBC – White Blood-cell Count, Low	6mo -5y	10^3/µL	5.1	16.2	2 - <5.1	1.5 - 1.999	1.0 - 1.499	<1.0
	6y - 10y	10^3/µL	4.5	12.9	2 - <4.5			
Neutrophils - Absolute Neutrophils Count, Low	6mo -5y	10^3/µL	1.3	6.9	0.8 - 1.299	0.6 - 0.799	0.4 - 0.599	<0.4
	6y - 10y	10^3/µL	1.2	6.2	0.8 - 1.199			
Lymphocytes - Absolute Lymphocytes Count, Low	6mo -5y	10^3/µL	2.1	9.5	0.6 - <2.1	0.5 - <0.6	0.35 - <0.5	<0.35
	>5y - 10y	10^3/µL	1.8	6.4	0.6 - <1.8			
Eosinophils - Absolute Eosinophil Count, High	6mo -5y	10^3/µL	0.1	1.6	>1.6 - 2.4	>2.4 - 4.8	> 4.8	*Hyper-eosinophilia*
	6y - 10y	10^3/µL	0.1	2.1	>2.1-3.1	>3.2 - 6.3	> 6.3	

**NORMAL RANGES AND TOXICITY GRADING FOR CLINICAL LABORATORY PARAMETERS; HEALTHY ADULT AND ADOLESCENT VOLUNTEERS (11-65 YEARS OLD)**						
**Biochemistry Parameters**	Units	Normal range	Mild (Grade1)	Moderate (Grade 2)	Severe (Grade 3)	Potentially life-threatening Grade (4)
Glucose – Hypoglycemia	mg/dL	70.3 - 109.8	64.9 - 70.2	55.9 - 64.8	45.05 - 55.8	<45.05
Creatinine	mg/dL	0.54 - 1.11	1.12 - 1.70	1.71 - 2.0	2.01 - 2.5	> 2.5 or require dialysis
Alanine aminotransferase	U/L	0 - 45	>45.1 – 106.5	106.6 – 209	>209.1 - 410	>410
Aspartate aminotransferase	U/L	15.2 – 58.7	58.8 - 138.8	138.9 - 272.3	272.4 - 534	>534
Bilirubin – when accompanied by any increase in Liver Enzymes	U/L	0 – 2.62	>31.1 - 38.9	>38.9 - 46.7	>46.7 - 54.4	>54.4
Bilirubin – when Liver Enzymes are normal	mg/dL	0 - 2.62	2.63 - 3.01	3.02 - 3.61	3.62 - 4.19	>4.19

**NORMAL RANGES AND TOXICITY GRADING FOR CLINICAL LABORATORY PARAMETERS; HEALTHY ADULT AND ADOLESCENT VOLUNTEERS (11-65 YEARS OLD)**						
**Hematology Parameters**	Units	Normal range	Mild (Grade1)	Moderate (Grade 2)	Severe (Grade 3)	Potentially life Threatening Grade (4)
Hemoglobin (Male)	g/dL	12 - 17.4	11.0 -11.9	9.5 – 10.9	8.0 – 9.4	< 8.0
Hemoglobin (Female)	g/dL	9.6 - 14.1	<9.6 – 8.0	<8.0 – 7.0	<7.0 – 4.0	<4.0
Leukocyte count (WBC) Decrease	$10^3/\mu L$	3.65 - 9.7	2.5 - <3.65	1.5 - 2.49	1 - 1.49	< 1
Lymphocytes, Decreased	$10^3/\mu L$	1.19 - 3.4	0.75 - <1.19	0.5 - 0.749	0.25 - 0.499	< 0.25
Neutrophils, Decreased	$10^3/\mu L$	1.61-5.69	0.9 - 1.60	0.6 - 0.89	0.4 - 0.59	<0.4
Eosinophils, Increased	$10^3/\mu L$	0 - 0.78	> 0.78 - 1.5	1.501 - 5	> 5	Hypereosinophilic
Platelets, Decreased	$10^3/\mu L$	124-312	75 - <124	50 - <75	25 – <50	< 25
Erythrocyte count (RBC)	$10^6/\mu L$	3.8 – 5.67	∞	∞	∞	∞

Table S4: Participant Demographic Data

		Group 1 (age 18-35 years)		Group 2 (age 36-61 years)		Group 3 (age 11-17 years)	
		**$2.7x10^6$ (N=20)**	**Placebo (N=6)**	**$2.7x10^6$ (N=12)**	**Placebo (N=3)**	**$1.8x10^6$ (N=12)**	**Placebo (N=4)**
Age	Units	Years	Years	Years	Years	Years	Years
	Mean (SD)	22.3 (3.8)	26.8 (5.9)	47.1 (8.4)	46.7 (9.3)	14.2 (2.1)	14.5 (2.5)
	Median	22	26	43	44	15	15
	(Min, Max)	(18,31)	(21,34)	(39,61)	(39,57)	(11,17)	(11,17)
Sex	Male	18 (90.0%)	5 (83.3%)	6 (50.0%)	2 (66.7%)	8 (66.7%)	2 (50.0%)
	Female	2 (10.0%)	1 (16.7%)	6 (50.0%)	1 (33.3%)	4 (33.3%)	2 (50.0%)
Race	African	20 (100.0%)	6 (100.0%)	12 (100.0%)	3 (100.0%)	12 (100.0%)	4 (100.0%)
Height (cm)	Mean (SD)	167.6 (9.2)	171.8 (8.6)	161.0 (5.0)	163.7 (11.4)	153.5 (13.7)	151.0 (19.2)
	Median	169.5	171.0	161.0	167.0	157.5	146.0
	(Min, Max)	(143,177)	(161,182)	(150,169)	(151,173)	(132,170)	(135,177)
Weight (kg)	Mean (SD)	67.4 (12.7)	72.5 (8.4)	65.3 (6.5)	66.2 (5.0)	49.0 (12.3)	46.5 (12.3)
	Median	67.3	72.6	66.6	65.0	52.8	46.3
	(Min, Max)	(42,90)	(62,85)	(52,73)	(62,72)	(31,68)	(33,60)
BMI	Mean (SD)	23.8 (3.2)	24.6 (2.5)	25.2 (2.5)	24.9 (3.2)	20.6 (3.9)	20.1 (1.8)
	Median	24.1	24.5	25.8	24.0	20.2	20.0
	(Min, Max)	(19,30)	(22,28)	(20,28)	(22,29)	(16,30)	(18,22)

		Group 4 (age 6-10 years)		Group 5 (age 1-5 years)		Group 6A (age 6-11 months)	Group 6B (age 6-11 months)	
		**$1.8x10^6$ (N=12)**	**Placebo (N=4)**	**$1.8x10^6$ (N=11)**	**Placebo (N=4)**	**$9.0x10^5$ (N=3)**	**$1.8x10^6$ (N=12)**	**Placebo (N=3)**
Age	Units	Years	Years	Years	Years	Months	Months	Months
	Mean (SD)	7.9 (1.2)	7.8 (0.5)	3.1 (1.3)	2.8 (1.0)	8.3 (1.5)	8.8 (1.9)	10.3 (0.6)
	Median	8	8	3	3	8	10	10
	(Min, Max)	(6,10)	(7,8)	(1,5)	(2,4)	(7,10)	(6,11)	(10,11)
Sex	Male	4 (33.3%)	1 (25.0%)	6 (54.5%)	3 (75.0%)	2 (66.7%)	8 (66.7%)	1 (33.3%)
	Female	8 (66.7%)	3 (75.0%)	5 (45.5%)	1 (25.0%)	1 (33.3%)	4 (33.3%)	2 (66.7%)
Race	African	12 (100.0%)	4 (100.0%)	11 (100.0%)	4 (100.0%)	3 (100.0%)	12 (100.0%)	3 (100.0%)
Height (cm)	Mean (SD)	127.5 (6.8)	123.3 (2.9)	92.3 (12.9)	88.0 (10.7)	67.0 (5.2)	64.5 (4.4)	64.7 (0.6)
	Median	127.3	123.0	91.0	85.5	70.0	62.5	65.0
	(Min, Max)	(116,141)	(120,127)	(68,110)	(78,103)	(61,70)	(60,74)	(64,65)
Weight (kg)	Mean (SD)	27.6 (6.3)	23.1 (1.9)	15.3 (4.0)	12.5 (2.5)	7.5 (0.6)	8.4 (1.0)	8.2 (0.6)
	Median	26.1	22.9	15.3	12.3	7.2	8.6	8.0
	(Min, Max)	(22,41)	(21,26)	(7,20)	(10,16)	(7,8)	(6,10)	(8,9)
BMI	Mean (SD)	16.8 (2.2)	15.2 (1.5)	N/A	N/A	N/A	N/A	N/A
	Median	16.7	15.1	N/A	N/A	N/A	N/A	N/A
	(Min, Max)	(14,22)	(14,17)	N/A	N/A	N/A	N/A	N/A
MUAC (cm)	Mean (SD)	N/A	N/A	16.0 (1.4)	14.8 (1.3)	14.0 (1.0)	14.9 (1.1)	14.2 (0.3)
	Median	N/A	N/A	15.8	15.0	14.0	15.0	14.2
	(Min, Max)	N/A	N/A	(14,18)	(13,16)	(13,15)	(13,17)	(14,15)

Table S5: Screen failures. Eighty-two individuals failed to satisfy 1 or more inclusion or exclusion criteria.

**Inclusion or exclusion criterion**	**Number of individuals not meeting the specified criteria**					
	**Group 1 Age 18-35 years**	**Group 2 Age 36-61 years**	**Group 3 Age 11-17 years**	**Group 4 Age 6-10 years**	**Group 5 Age 1-5 years**	**Group 6 Age 6-11 months**
History of psychiatric illness		1				
History of chronic disease (diabetes, malignancy, cardiovascular disease)	1	12				
Active drug or alcohol use		1				
Immunosuppressive drug use		1				
Positive testing for HIV, HBV or HCV	12	1	2	1		
Evidence of clinically active tuberculosis	1	1				
History of afebrile seizures or atypical febrile seizures						1
Unable to comply with study schedule		2				
Unable to provide informed consent	1					
Pregnant at the time of screening or unwilling to provide consent/assent to not become pregnant during the study	6	1	2			
BMI < 18 or > 30 kg/m^2 or comparable Z-score for adolescents and children	7	8	1			1
Positive for malaria		1				
Positive for helminth infection	2	1			2	
Did not meet the definition of healthy as judged by clinical and laboratory findings	2	5			1	4

Table S6: Injection Pain, Efficiency and Speed

		Group 1 (age 18-35 years)		Group 2 (age 36-61 years)		Group 3 (age 11-17 years)	
		**$2.7x10^6$ All Injections (N=56)**	**Placebo All Injections (N=18)**	**$2.7x10^6$ All Injections (N=32)**	**Placebo All Injections (N=9)**	**$1.8x10^6$ All Injections (N=35)**	**Placebo All Injections (N=12)**
Total number of syringes	1	56 (100.0%)	18 (100.0%)	32 (100.0%)	9 (100.0%)	35 (100.0%)	12 (100.0%)
	2	0 (0%)	0 (0%)	0 (0%)	0 (0%)	0 (0%)	0 (0%)
Volunteer pain assessment	1 - Painless	26 (46.4%)	10 (55.6%)	10 (31.3%)	5 (55.6%)	19 (54.3%)	4 (33.3%)
	2 - Mild pain	25 (44.6%)	7 (38.9%)	22 (68.8%)	3 (33.3%)	13 (37.1%)	8 (66.7%)
	3 - Moderate pain	3 (5.4%)	1 (5.6%)	0 (0%)	0 (0%)	1 (2.9%)	0 (0%)
	4 - Severe pain	1 (1.8%)	0 (0%)	0 (0%)	1 (11.1%)	2 (5.7%)	0 (0%)
	Missing	1 (1.8%)	0 (0%)	0 (0%)	0 (0%)	0 (0%)	0 (0%)
Total number of injection attempts (stick injections)	1	54 (96.4%)	18 (100.0%)	31 (96.9%)	9 (100.0%)	33 (94.3%)	9 (75.0%)
	2	2 (3.6%)	0 (0%)	1 (3.1%)	0 (0%)	2 (5.7%)	3 (25.0%)
	3	0 (0%)	0 (0%)	0 (0%)	0 (0%)	0 (0%)	0 (0%)
	4 or more sticks	0 (0%)	0 (0%)	0 (0%)	0 (0%)	0 (0%)	0 (0%)

		Group 4 (age 6-10 years)		Group 5 (age 1-5 years)		Group 6A (age 6-11 months)	Group 6B (age 6-11 months)	
		**1.8x10^6 All Injections (N=33)**	**Placebo All Injections (N=12)**	**1.8x10^6 All Injections (N=31)**	**Placebo All Injections (N=9)**	**9.0x10^5 All Injections (N=3)**	**1.8x10^6 All Injections (N=35)**	**Placebo All Injections (N=9)**
Total number of syringes	1	31 (93.9%)	12 (100.0%)	28 (90.3%)	9 (100.0%)	3 (100.0%)	34 (97.1%)	9 (100.0%)
	2	2 (6.1%)	0 (0%)	3 (9.7%)	0 (0%)	0 (0%)	1 (2.9%)	0 (0%)
Volunteer pain assessment	1 - Painless	15 (45.5%)	3 (25.0%)	N/A	N/A	N/A	N/A	N/A
	2 - Mild pain	16 (48.5%)	7 (58.3%)	N/A	N/A	N/A	N/A	N/A
	3 - Moderate pain	1 (3.0%)	1 (8.3%)	N/A	N/A	N/A	N/A	N/A
	4 - Severe pain	1 (3.0%)	1 (8.3%)	N/A	N/A	N/A	N/A	N/A
Did the child cry?	Yes	1 (3.0%)	0 (0%)	18 (58.1%)	8 (88.9%)	3 (100.0%)	29 (82.9%)	8 (88.9%)
	No	32 (97.0%)	12 (100.0%)	12 (38.7%)	1 (11.1%)	0 (0%)	5 (14.3%)	0 (0%)
	N/A	0 (0%)	0 (0%)	1 (3.2%)	0 (0%)	0 (0%)	1 (2.9%)	0 (0%)
	Missing	0 (0%)	0 (0%)	0 (0%)	0 (0%)	0 (0%)	0 (0%)	1 (11.1%)
If Yes, cry at what?	After	0 (0%)	0 (0%)	2 (6.5%)	2 (22.2%)	1 (33.3%)	25 (71.4%)	6 (66.7%)
	Before	1 (3.0%)	0 (0%)	15 (48.4%)	5 (55.6%)	3 (100.0%)	17 (48.6%)	6 (66.7%)
	During	0 (0%)	0 (0%)	13 (41.9%)	7 (77.8%)	2 (66.7%)	29 (82.9%)	7 (77.8%)
Total number of injection attempts (stick injections)	1	31 (93.9%)	12 (100.0%)	26 (83.9%)	8 (88.9%)	3* (100.0%)	28 (80.0%)	7 (77.8%)
	2	2 (6.1%)	0 (0%)	3 (9.6%)	1 (11.1%)	0 (0%)	5 (14.3%)	1 (11.1%)
	3	0 (0%)	0 (0%)	2 (6.5%)	0 (0%)	0 (0%)	2 (5.7%)	1 (11.1%)
	4 or more sticks	0 (0%)	0 (0%)	0 (0%)	0 (0%)	0 (0%)	0 (0%)	0 (0%)

N is number of vaccination records submitted.

If more than one syringe was used, data from all attempts with all syringes are included in this table. Insertion of a catheter is counted as 1 injection attempt.

* includes 2 catheter insertions without attempt at DVI

Table S7. Unsolicited adverse events possibly, probably or definitely related to investigational product (IP).

Study Group (Age Group)	Treatment Group	Sex	Days Elapsed from Prior Vaccination	Adverse Event	MedDRA System Organ Class/ Preferred Term	Duration (Days)	Severity	Relationship to Investigational Product	Alternative Etiology	Outcome
1 (18-35 years)	$2.7x10^6$	Male	5	ACUTE GASTRITIS	Gastrointestinal disorders/ Gastritis	2	Moderate	Probably related	Other medical condition or illness: Unknown	Recovered/ resolved
2 (36-61 years)	$2.7x10^6$	Female	13	DIARRHEA	Gastrointestinal disorders/ Diarrhoea	7	Mild	Possibly related	Other medical condition or illness: Gastroenteritis	Recovered/ resolved
3 (11-17 years)	$1.8x10^6$	Male	0	NON-FEBRILE GENERALIZED TONIC CLONIC SEIZURES	Nervous system disorders/ Generalized tonic-clonic seizure	1	Mild	Possibly related	Other medical condition or illness: Genetic predisposition	Recovered/ resolved
3 (11-17 years)	$1.8x10^6$	Male	3	ABDOMINAL PAIN	Gastrointestinal disorders/ Abdominal pain	2	Mild	Possibly related	Other medical condition or illness: Unknown	Recovered/ resolved
4 (6-10 years)	$1.8x10^6$	Female	12	SUBJECTIVE FEVER	General disorders and administration site conditions/ Pyrexia	1	Mild	Possibly related	Other medical condition or illness: Febrile syndrome	Recovered/ resolved
5 (1-5 years)	$1.8x10^6$	Female	11	ALLERGIC DERMATITIS	Skin and subcutaneous tissue disorders/ Dermatitis allergic	7	Moderate	Possibly related	Other: Possible allergy	Recovered/ resolved
5 (1-5 years)	Placebo	Male	21	FEVER	General disorders and administration site conditions/ Pyrexia	6	Moderate	Possibly related	Other medical condition or illness: Viral infection	Recovered/ resolved
6b (6-11 months)	Placebo	Female	7	DERMATITIS	Skin and subcutaneous tissue disorders/ Dermatitis	3	Moderate	Possibly related	Study procedure: Ambient temperature	Recovered/ resolved

Table S8. Laboratory abnormalities post immunization. Only lymphopenia was considered possibly related to immunization. Abnormal Lab Values are defined as Grade 1 or higher per the protocol defined toxicity ranges. Table includes all post-vaccination results.

	**Group 1 (age 18-35 years)**		**Group 2 (age 36-61 years)**		**Group 3 (age 11-17 years)**	
**Lab parameter**	**$2.7x10^6$ (N=20)**	**Placebo (N=6)**	**$2.7x10^6$ (N=12)**	**Placebo (N=3)**	**$1.8x10^6$ (N=12)**	**Placebo (N=4)**
Red Blood Cells	0 (0.0)	0 (0.0)	0 (0.0)	0 (0.0)	0 (0.0)	0 (0.0)
Decreased Hemoglobin	2 (10.0)	2 (33.3)	1 (8.3)	0 (0.0)	4 (33.3)	1 (25.0)
Decreased Platelets	2 (10.0)	1 (16.7)	0 (0.0)	1 (33.3)	0 (0.0)	0 (0.0)
Increased WBC Count	0 (0.0)	0 (0.0)	0 (0.0)	0 (0.0)	1 (8.3)	0 (0.0)
Decreased WBC Count	7 (35.0)	3 (50.0)	4 (33.3)	2 (66.7)	4 (33.3)	2 (50.0)
Decreased Neutrophils	15 (75.0)	4 (66.7)	11 (91.7)	2 (66.7)	9 (75.0)	3 (75.0)
Decreased Lymphocytes	3 (15.0)	2 (33.3)	3 (25.0)*	1 (33.3)	2 (16.7)	0 (0.0)
Increased Eosinophils	7 (35.0)	3 (50.0)	2 (16.7)	0 (0.0)	6 (50.0)	2 (50.0)
Elevated ALT	2 (10.0)	3 (50.0)	4 (33.3)	0 (0.0)	0 (0.0)	0 (0.0)
Elevated AST	3 (15.0)	1 (16.7)	2 (16.7)	0 (0.0)	1 (8.3)	0 (0.0)
Elevated Total Bilirubin	0 (0.0)	0 (0.0)	0 (0.0)	0 (0.0)	0 (0.0)	0 (0.0)
Elevated Creatinine	4 (20.0)	1 (16.7)	3 (25.0)	0 (0.0)	0 (0.0)	0 (0.0)
Hypoglycemia	0 (0.0)	0 (0.0)	0 (0.0)	0 (0.0)	0 (0.0)	0 (0.0)

	Group 4 (age 6-10 years)		Group 5 (age 1-5 years)		Group 6a (age 6-11 months)	Group 6b (age 6-11 months)	
**Lab parameter**	**$1.8x10^6$ (N=12)**	**Placebo (N=4)**	**$1.8x10^6$ (N=11)**	**Placebo (N=4)**	**$9.0x10^5$ (N=3)**	**$1.8x10^6$ (N=12)**	**Placebo (N=3)**
Red Blood Cells	0 (0.0)	0 (0.0)	0 (0.0)	0 (0.0)	0 (0.0)	0 (0.0)	0 (0.0)
Decreased Hemoglobin	2 (16.7)	0 (0.0)	2 (18.2)	1 (25.0)	0 (0.0)	5 (41.7)	0 (0.0)
Decreased Platelets	0 (0.0)	0 (0.0)	0 (0.0)	0 (0.0)	0 (0.0)	2 (16.7)*	0 (0.0)
Increased WBC Count	0 (0.0)	0 (0.0)	0 (0.0)	0 (0.0)	0 (0.0)	0 (0.0)	0 (0.0)
Decreased WBC Count	3 (25.0)	0 (0.0)	3 (27.3)	1 (25.0)	0 (0.0)	0 (0.0)	0 (0.0)
Decreased Neutrophils	2 (16.7)	0 (0.0)	3 (27.3)	1 (25.0)	0 (0.0)	2 (16.7)	0 (0.0)
Decreased Lymphocytes	2 (16.7)	0 (0.0)	5 (45.5)	1 (25.0)	0 (0.0)	0 (0.0)	0 (0.0)
Increased Eosinophils	0 (0.0)	2 (50.0)	5 (45.5)	2 (50.0)	0 (0.0)	5 (41.7)	3 (100.0)
Elevated ALT	4 (33.3)	2 (50.0)	5 (45.5)*	2 (50.0)	1 (33.3)	6 (50.0)*	2 (66.7)*
Elevated AST	4 (33.3)	1 (25.0)	3 (27.3)*	0 (0.0)	0 (0.0)	3 (25.0)	1 (33.3)*
Elevated Total Bilirubin	0 (0.0)	0 (0.0)	0 (0.0)	0 (0.0)	0 (0.0)	0 (0.0)	0 (0.0)
Elevated Creatinine	0 (0.0)	0 (0.0)	0 (0.0)	0 (0.0)	0 (0.0)	0 (0.0)	0 (0.0)
Hypoglycemia	0 (0.0)	0 (0.0)	0 (0.0)	0 (0.0)	0 (0.0)	0 (0.0)	0 (0.0)

*Includes at least one Grade 3 result.

Table S9: Descriptions of grade 3 laboratory abnormalities.

1. A 10-month-old girl receiving NS developed persistent ALT and AST elevations 6 weeks after the initial screening visit. Her clinical and laboratory course is provided in detail in the clinical summaries section.
2. A 7-month-old girl receiving 1.8×10^6 PfSPZ of PfSPZ Vaccine developed isolated grade 3 elevation of ALT (151.9 U/L) 14 days after the 3rd dose. Results 2 days and 50 days after the 3rd dose were normal; at other time points results ranged from normal to grade 2 elevations. This same participant had isolated grade 3 thrombocytopenia (46,000/µL) 55 days after the first vaccine dose; all other platelet counts were normal.
3. A 2-year-old male receiving 1.8×10^6 PfSPZ of PfSPZ Vaccine developed isolated grade 3 ALT (125.4 U/L) and grade 4 AST (795.2 U/L) elevations 14 days after the second dose. All other measurements of ALT and AST were normal for this child, including ALT and AST drawn 2, 13 or 14 days after the first and 3rd doses.
4. An 11-month-old male receiving 1.8×10^6 PfSPZ of PfSPZ Vaccine developed isolated grade 3 thrombocytopenia (34,000/µL) 10 days after the 3rd vaccine dose. Results obtained 2 days and 14 days after the 3rd dose were 400,000/µL and 561,000/µL, respectively, suggesting this result was due to laboratory error.
5. A 44-year-old male receiving 2.7×10^6 PfSPZ of PfSPZ Vaccine developed grade 3 lymphopenia (370/µL) 1 day after the second vaccine dose. All other lymphocyte counts were normal, including results 2 days after the 1st and 3rd dose.

Table S10: Asymptomatic parasitemia retrospectively detected by qPCR. Prior to CHMI (Group 1) or at the end of the study, samples were tested using a specific *P. falciparum* qPCR and a pan-plasmodium 18s RNA PCR. Eight study participants were found to have 1 or more asymptomatic *Plasmodium* sp. infections during the specified interval. All participants were treated for the identified infections at the time of detection.

Group	Vaccine	Time point(s)	Species
1	$2.7x10^6$ PfSPZ	Screening	*P. malariae*
		V2	*P. falciparum*
		CHMI-7	*P. falciparum*
		CHMI	*P. falciparum, P. malariae*
1	placebo	V3, V3+28, V3+56	*P. malariae*
1	$2.7x10^6$ PfSPZ	V3	*P. ovale*
		V3+196	*P. falciparum*
1	placebo	Scr3	*P. falciparum, P. malariae*
		V2	*P. malariae*
		V3, V3+28, V3+56	*P. falciparum*
2	$2.7x10^6$ PfSPZ	V3+112	*P. falciparum*
2	$2.7x10^6$ PfSPZ	V2, V3, V3+84	*P. falciparum*
2	$2.7x10^6$ PfSPZ	V3, V3+28, V3+56, V3+112, V3+168	*P. malariae*
2	$2.7x10^6$ PfSPZ	Sc3, V2, V3+112, V3+168	*P. malariae*

Table S11: Antibodies against PfCSP measured by ELISA in human sera from EGSPZV2 study; measured pre-immunization, 2 weeks post 3rd dose of PfSPZ Vaccine and pre-CHMI (only for 18-25-year-olds). All out-of-range values, negatives and zeroes are reported as 1. 1 = baseline sample was not available; value reported as 1 for calculation of net and ratio OD 1.0.

Age	PfSPZ/Dose	Volunteer ID	ELISA PfCSP OD 1.0						
			Pre-Immunization	2 weeks post-3rd dose	NET (Post-Pre)	Ratio (Post/Pre)	pre-CHMI	NET pre-CHMI (Post-Pre)	Ratio (Post/Pre)
36-61 years old	2.7x10^{6} PfSPZ Vaccine	E202-304	41	10,102	10,061	246	N/A		
		E202-305	92	44,042	43,950	479			
		E202-339	64	1,947	1,883	30			
		E202-346	62	3,180	3,118	51			
		E202-351	260	19,383	19,123	75			
		E202-476	795	7,237	6,442	9			
		E202-497	308	3,803	3,495	12			
		E202-501	322	701	379	2			
		E202-502	429	191	1	1			
**Median**			**260**	**3,803**	**3,495**	**30**			
36-61 years old	Placebo	E202-310	100	69	1	1			
		E202-424	58	47	1	1			
**Median**			**79**	**58**	**1**	**1**			
18-35 years old	2.7x10^{6} PfSPZ Vaccine	E21A317	30	2,966	2,936	99	2,182	2,152	73
		E21A371	198	5,797	5,599	29	3,575	3,377	18
		E21A412	130	2,358	2,228	18	1,615	1,485	12
		E21A414	319	11,569	11,250	36	15,892	15,573	50
		E21A416	NS1	2,911	2,911	2,911	2,494	2,494	2,494
		E21A444	17	5,867	5,850	345	4,014	3,997	236
		E21A309	39	5,021	4,982	129	5,442	5,403	140
		E21A311	108	4,001	3,893	37	1,094	986	10
		E21A313	224	14,587	14,363	65	4,504	4,280	20
		E21A314	73	2,261	2,188	31	520	447	7
		E21A316	21	2,052	2,031	98	1,027	1,006	49
		E21A399	1	2,601	2,600	2,601	1,057	1,056	1,057
		E21A402	55	599	544	11	320	265	6
		E21A408	892	4,017	3,125	5	3,041	2,149	3
		E21A417	1,363	4,499	3,136	3	2,381	1,018	2
		E21A426	958	6,474	5,516	7	NS	-	-
		E21A433	31	1,251	1,220	40	1,359	1,328	44
		E21A448	7	909	902	130	384	377	55
**Median**			**73**	**3,484**	**3,031**	**39**	**2,182**	**1,485**	**44**
18-35 years old	Placebo	E21A411	979	814	813	1.00	559	558	1
		E21A422	108	95	1	1.00	147	39	1
		E21A303	64	44	1	1.00	57	1	1
		E21A431	254	294	40	1.16	300	46	1
		E21A472	18	22	4	1.22	31	13	2
**Median**			**108**	**95**	**4**	**1**	**147**	**39**	**1**

Age	PfSPZ/Dose	Volunteer ID	ELISA PfCSP OD 1.0						
			Pre-Immunization	2 weeks post-3rd dose	NET (Post-Pre)	Ratio (Post/Pre)	pre-CHMI	NET pre-CHMI (Post-Pre)	Ratio (Post/Pre)
11-17 years old	$1.8x10^6$ PfSPZ Vaccine	E203-319	70	2,055	1,985	29	N/A		
		E203-320	61	6,730	6,669	110			
		E203-347	6	4,148	4,142	691			
		E203-349	1	15,393	15,392	15,393			
		E203-350	11	16,097	16,086	1,463			
		E203-352	86	4,554	4,468	53			
		E203-362	26	9,472	9,446	364			
		E203-365	12	16,857	16,845	1,405			
		E203-370	37	6,994	6,957	189			
		E203-386	27	13,656	13,629	506			
		E203-460	66	4,094	4,028	62			
**Median**			**27**	**6,994**	**6,957**	**364**			
11-17 years old	Placebo	E203-338	19	45	26	2			
		E203-359	46	52	6	1			
		E203-390	173	162	1	1			
		E203-442	20	20	0	1			
**Median**			**33**	**49**	**4**	**1**			
6-10 years old	$1.8x10^6$ PfSPZ Vaccine	E204-321	26	9,159	9,133	352			
		E204-337	59	18,827	18,768	319			
		E204-354	22	7,545	7,523	343			
		E204-364	12	8,622	8,610	719			
		E204-375	12	5,831	5,819	486			
		E204-383	18	19,561	19,543	1,087			
		E204-419	16	10,744	10,728	672			
		E204-463	48	3,803	3,755	79			
		E204-468	10	16,922	16,912	1,692			
		E204-471	27	9,424	9,397	349			
**Median**			**20**	**9,292**	**9,265**	**419**			
6-10 years old	Placebo	E204-322	26	71	45	3			
		E204-355	1	16	15	16			
		E204-420	20	39	19	2			
		E204-490	14	1	1	1			
**Median**			**17**	**28**	**17**	**2**			
1-5 years old	$1.8x10^6$ PfSPZ Vaccine	E205-489	NS1	116,903	116,902	116,903			
		E205-540	NS1	17,400	17,399	17,400			
		E205-553	6	59,295	59,289	9,883			
		E205-554	59	9,403	9,344	159			
		E205-557	36	5,330	5,294	148			
		E205-558	1	25,384	25,383	25,384			
		E205-559	24	15,024	15,000	626			
		E205-561	47	33,913	33,866	722			
		E205-566	76	5,619	5,543	74			
		E205-568	82	12,701	12,619	155			
**Median**			**42**	**16,212**	**16,200**	**674**			
6-11	$1.8x10^6$	E26B-584	23	2,712	2,689	118			

Age	PfSPZ/Dose	Volunteer ID	ELISA PfCSP OD 1.0						
			Pre-Immunization	2 weeks post-3rd dose	NET (Post-Pre)	Ratio (Post/Pre)	pre-CHMI	NET pre-CHMI (Post-Pre)	Ratio (Post/Pre)
months old	PfSPZ Vaccine	E26B-585	1	5,731	5,730	5,731			
		E26B-587	1	14,417	14,416	14,417			
		E26B-588	NS1	5,921	5,920	5,921			
		E26B-590	NS1	37,959	37,958	37,959			
		E26B-593	44	27,441	27,440	624			
		E26B-595	1	190,309	190,308	190,309			
		E26B-596	59	12,262	12,203	208			
		E26B-597	333	45,063	44,730	135			
		E26B-598	10	7,357	7,347	736			
		E26B-599	30	17,944	17,914	598			
**Median**			**23**	**14,417**	**14,416**	**736**			
